# Supplementary material for: Early postnatal serotonin modulation prevents adult-stage deficits in Arid1b-deficient mice through synaptic transcriptional reprogramming
Source: Nat Commun. 2022 Aug 27;13:5051. doi: 10.1038/s41467-022-32748-5 (PMC9420115; doi:10.1038/s41467-022-32748-5)
Supplement: Supplementary file 1 — Supplementary Information [file 41467_2022_32748_MOESM1_ESM.pdf]

Early postnatal serotonin modulation prevents adult-stage deficits in *Arid1b*-deficient mice through synaptic transcriptional reprogramming

Hyosang Kim,[1,*] Doyoun Kim,[2,*] Yisul Cho,[3,*] Kyungdeok Kim,[2,] Junyeop Daniel Roh,[2] Yangsik Kim,[4] Esther Yang,[5] Seong Soon Kim,[6] Sunjoo Ahn,[6] Hyun Kim,[5] Hyojin Kang,[7] Yongchul Bae,[3,#] and Eunjoon Kim[1,2,#]

[1]Department of Biological Sciences, Korea Advanced Institute for Science and Technology (KAIST), Daejeon 34141, Korea; [2]Center for Synaptic Brain Dysfunctions, Institute for Basic Science (IBS), Daejeon 34141, Korea; [3]Department of Anatomy and Neurobiology, School of Dentistry, Kyungpook National University, Daegu 41940, Korea; [4]Graduate School of Biomedical Engineering, Korea Advanced Institute for Science and Technology (KAIST), Daejeon 34141, Korea; [5]Department of Anatomy and Division of Brain Korea 21, Biomedical Science, College of Medicine, Korea University, Seoul 02841, Korea; [6]Therapeutics and Biotechnology Division, Korea Research Institute of Chemical Technology (KRICT), Daejeon 34114, Korea; [7]Division of National Supercomputing, Korea Institute of Science and Technology Information, Daejeon 34141, Korea; *These authors contributed equally to the work; [#]Co-corresponding author.

Supplementary figures and legends

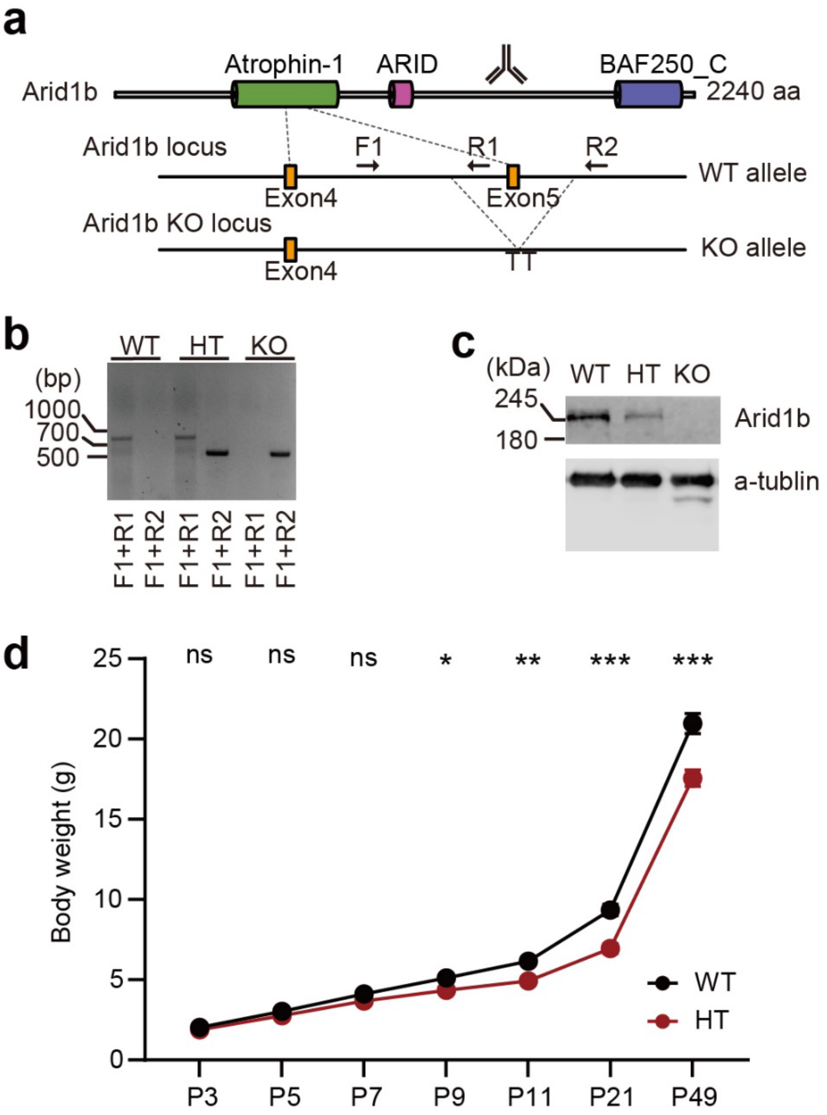

Supplementary Figure 1. Generation of *Arid1b*$^{+/-}$ mice.

(a) Strategy for *Arid1b* knockout (KO) in mice. A protein-truncating mutation was introduced by inserting “TT” nucleotides at the starting region of exon 5, which encodes a part of the atrophin-1 domain. PCR primer target regions (F1, R1, and R2), and antibody target regions are indicated. Atrophin-1, ARID, and BAF250_C indicate specific domains of the Arid1b protein.

(b) PCR genotyping of WT, *Arid1b*$^{+/-}$ (HT), and *Arid1b*$^{-/-}$ (KO) mice. Similar results were obtained from three independent experiments.

(c) Reduced and undetectable levels of ARID1B protein in *Arid1b*$^{+/-}$ (HT) and *Arid1b*$^{-/-}$ (KO) mice, respectively, at P0. See Figure 1b for quantification.

(d) Reduced body weights in *Arid1b*$^{+/-}$ mice across postnatal developmental stages (male and female mouse weights were combined). (n = 15 mice [male-WT], 17

[male-HT/heterozygote], 17 [female-WT], and 12 [female-HT], Sidak's multiple comparison test, [P3: $p = 0.2649$, P5: $p = 0.3043$, P7: $p = 0.1350$, P9: $p = 0.0154$, P11: $p = 0.0014$, P21: $p < 0.0001$, P49: $p = 0.0006$]).

Graphical data are presented as means ± SEM (*$p < 0.05$, **$p < 0.01$, ***$p < 0.001$, ns, not significant). Source data are provided as a Source Data file.

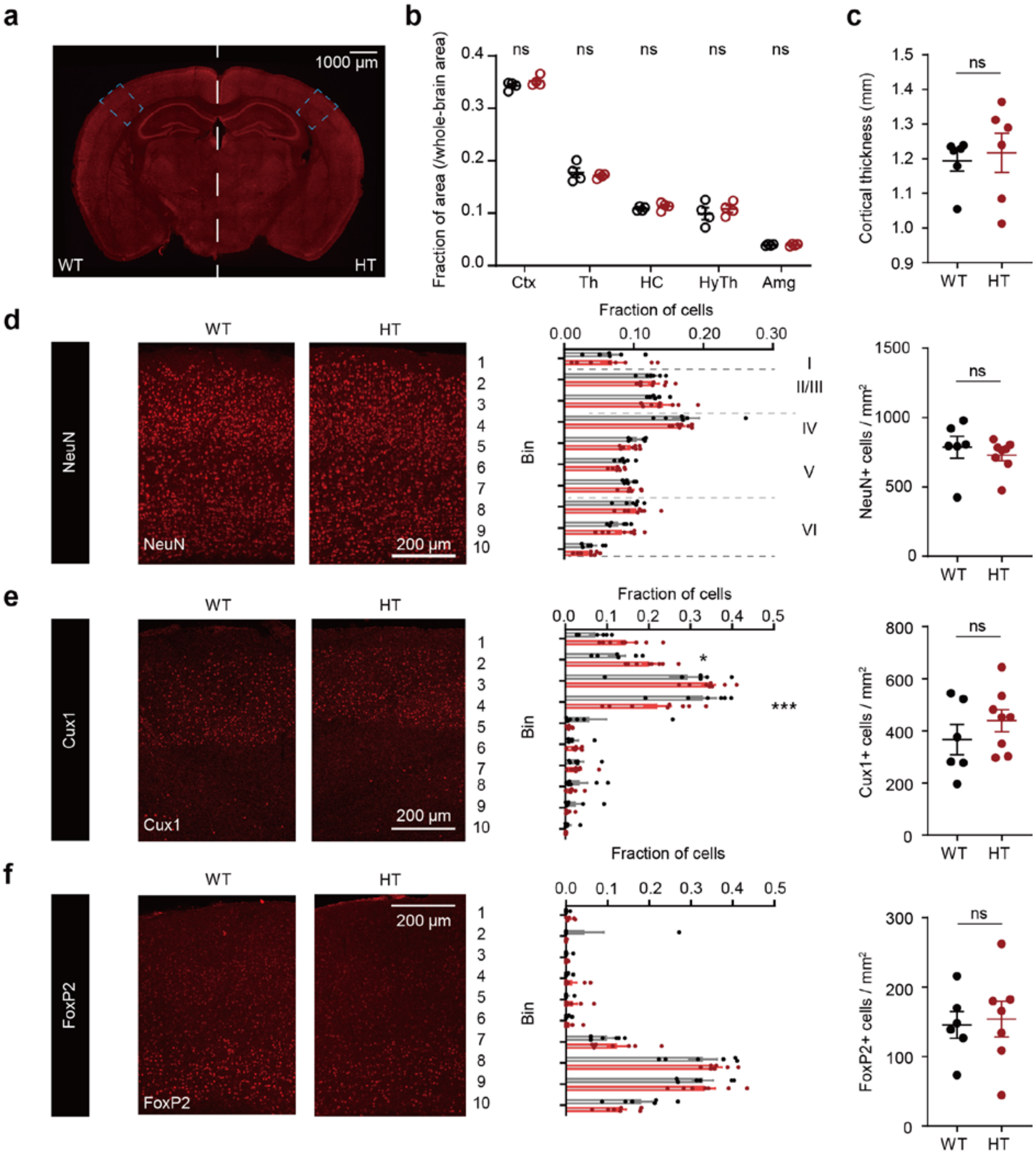

Supplementary Figure 2. Largely normal architecture of the *Arid1b*$^{+/-}$ brain.

(a–c) Normal sub-brain regional areas and cortical thicknesses (somatosensory cortex) in *Arid1b*$^{+/-}$ mice (P42), as shown by NeuN staining. Ctx, cortex; Th, thalamus; HC, hippocampus; HyTh, hypothalamus; Amg, Amygdala. (n = 6 slices from 4 mice [WT/wild-type and HT/heterozygous], two-tailed Student's t-test and two-tailed Mann-Whitney test [cortex thickness]).

(d–f) Largely normal cell numbers in total, upper, and lower cortical layers

(somatosensory cortex) in *Arid1b*$^{+/–}$ mice, revealed by staining for NeuN (RBFOXP3), CUX1, and FOXP2, respectively. Neuronal densities across cortical depths are shown by neuronal fractions. Note a moderate upward shift in CUX1-positive neurons in *Arid1b*$^{+/–}$ mice. (n = 6 slices from 4 mice [WT-NeuN], 8, 4 [HT-NeuN], 6, 2 [WT-Cux1], 8, 4 [HT-Cux1], 6, 3 [WT-FoxP2], and 7, 3 [HT-FoxP2], two-tailed Student's t-test [Cux1 and FoxP2] and two-tailed Mann-Whitney test [NeuN], two-way ANOVA with Sidak's test for fraction of cells. [Cux1, Bin 2: $p = 0.0441$, Bin 4: $p = 0.0006$]).

Graphical data are presented as means ± SEM (*$p < 0.05$, **$p < 0.01$, ***$p < 0.001$, ns, not significant). Source data are provided as a Source Data file.

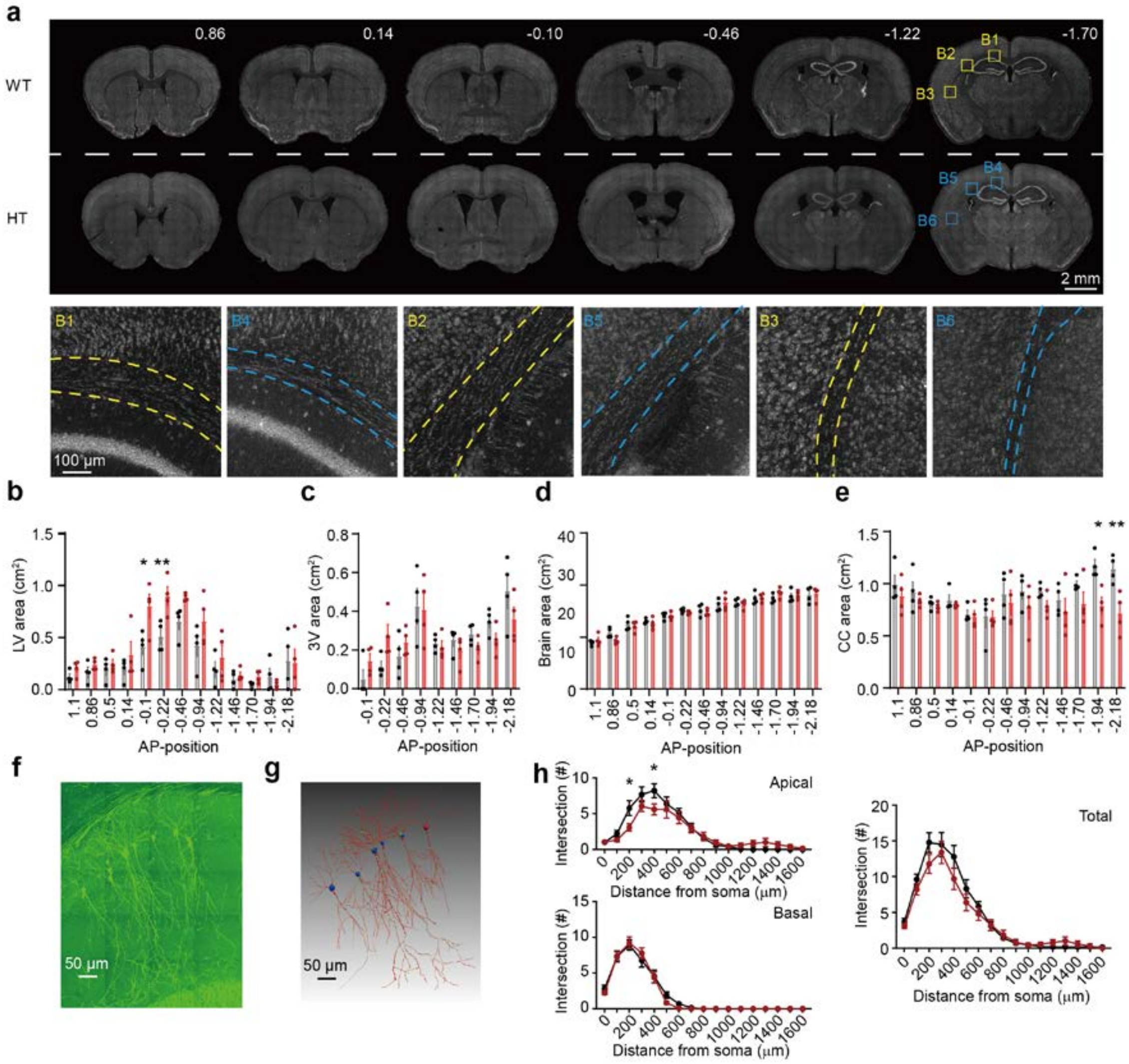

Supplementary Figure 3. Moderately altered ventricular and corpus callosal areas and hippocampal dendritic complexity in the *Arid1b*$^{+/-}$ brain.

(a–e) Moderate increases in the area of lateral ventricles (mid areas) and moderate decreases in the area of the corpus callosum (posterior areas). Note that areas of the third ventricle and whole brain are normal. Coronal brain sections from WT and *Arid1b*$^{+/-}$ mice (P42) were analyzed by Nissl staining. LV, lateral ventricle; 3V, third ventricle; CC, corpus callosum; A-P, anterior-posterior. (n = 4 mice [WT and HT], two-way ANOVA with Sidak's test, [Lateral Ventricle, -0.1: p = 0.0107, -0.22: p = 0.0095], [Corpus callosum, -1.94: p = 0.0108, -2.18: p = 0.0029]).

(f–h) Moderate decreases in the complexity of apical, but not basal, dendrites in CA1 hippocampal neurons in *Arid1b*$^{+/-}$;Thy1-EGFP mice (P42), as determined by Sholl analysis. (n = 13 neurons from 2 mice [WT], and 13, 3 [KO], two-way ANOVA with Sidak's test, [Apical, 200: p = 0.0124, 400: p = 0.0174]).

Graphical data are presented as means ± SEM (*p < 0.05, **p < 0.01, ***p < 0.001, ns, not significant). Source data are provided as a Source Data file.

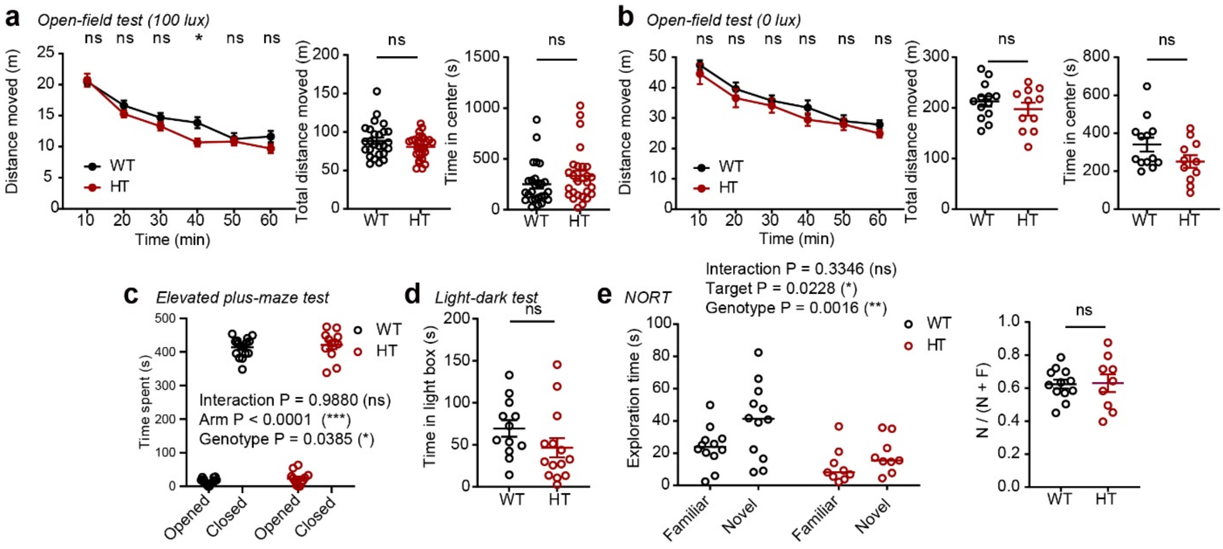

Supplementary Figure 4. Normal levels of locomotor activity, anxiety-like behavior, and object-recognition memory in *Arid1b*$^{+/–}$ mice.

(a and b) Normal levels of locomotor activity in *Arid1b*$^{+/–}$ mice (2–3 months) in the open-field test under a light intensity of 100 lux (a) and in complete darkness (b), as shown by distance moved. Note that the time spent in the center region of the open-field arena was normal in the mutant mice, suggestive of normal anxiety-like behavior. (n = 26 mice [WT-100 lux], 27 [HT-100 lux], 13 [WT-darkness], 11 [HT-darkness], two-way ANOVA with Sidak's test [distance moved], two-tailed Student's t-test [total distance moved, center time in darkness], and two-tailed Mann-Whitney test [total distance moved, center time in 100 lux], [100lux, 40 min: p = 0.0251]).

(c) Normal anxiety-like behavior in *Arid1b*$^{+/–}$ mice (2–3 months) in the elevated plus-maze test. (n = 16 [WT], 12 [HT], two-way ANOVA with Tukey's test, [Opened-WT vs Closed-WT: p < 0.0001, Opened-HT vs Closed-HT: p < 0.0001]).

(d) Normal anxiety-like behavior in *Arid1b*$^{+/–}$ mice (2–3 months) in the light-dark test. (n = 12 [WT], 14 [HT], ns, not significant, two-tailed Mann-Whitney test).

(e) Normal object memory in *Arid1b*$^{+/–}$ mice (2–3 months) in the novel object-recognition test (NORT), as shown by the ratio of time spent exploring familiar and novel (F/N) objects and the ratio of novel-target/total exploration. (n = 12 [WT], 9 [HT], two-way ANOVA with Tukey's test [F/N exploration], two-tailed Mann-Whitney test [ratio of N/[F+N]]).

Graphical data are presented as means ± SEM (*p < 0.05, **p < 0.01, ***p < 0.001, ns, not significant). Source data are provided as a Source Data file.

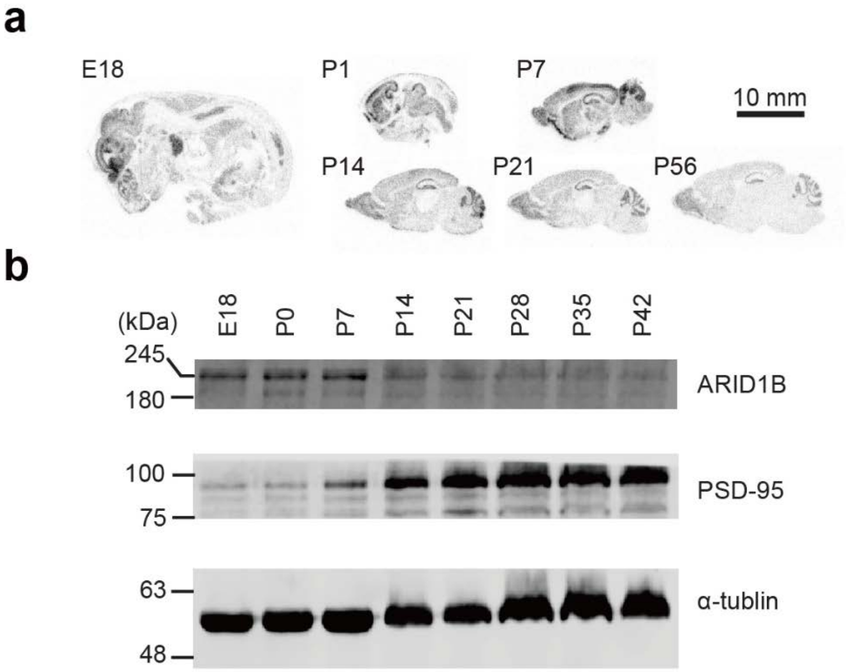

Supplementary Figure 5. Distribution patterns of Arid1b mRNAs and ARID1B proteins in the wild type developing mouse brain.

(a) In situ hybridization analysis of the distribution patterns of *Arid1b* mRNA in WT mouse brains at E18, P1, P7, P14, P21 and P56 using a radioisotope probe. E, embryonic day; P, postnatal day.

(b) Expression levels of ARID1B protein in WT mouse brains at E18, P0, P7, P14, P21, P28, P35, and P42, as determined by immunoblot analysis of whole-brain lysates. PSD-95, an abundant excitatory postsynaptic density protein, is shown for comparison. The full-length blot was divided into three parts for immunoblotting with different antibodies. Similar results were obtained from three independent experiments.

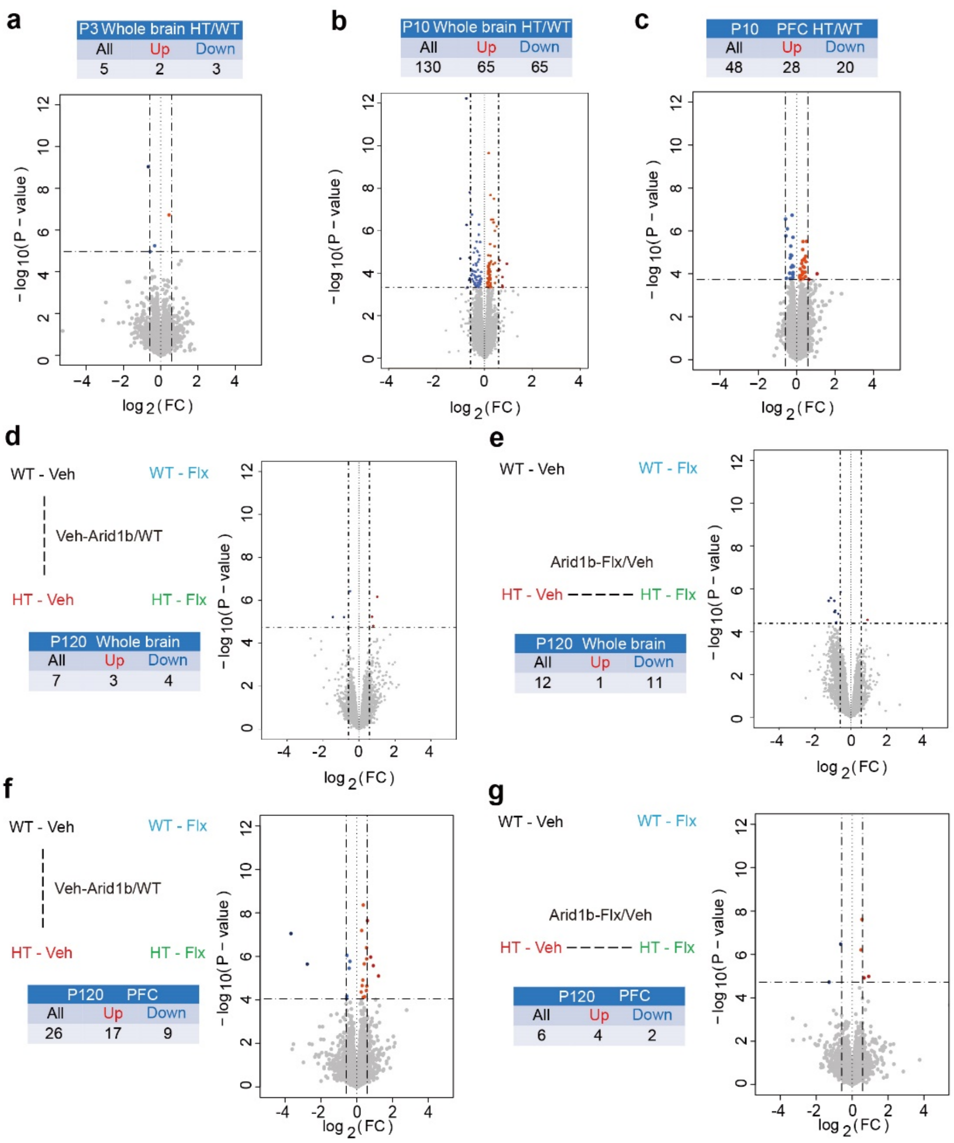

Supplementary Figure 6. Volcano plots of Arid1b/WT transcripts from P3/10 whole brain, P10 mPFC, and P120 whole brain and mPFC.

(a) Volcano plots of DEGs (FDR < 0.05) in Arid1b/WT transcripts from P3 whole brain. P, postnatal. (n = 5 [WT], 5 [HT]).

(b) Volcano plots of DEGs (FDR < 0.05) in Arid1b/WT transcripts from P10 whole brain. (n = 5 [WT], 5 [HT]).

(c) Volcano plots of DEGs (FDR < 0.05) in Arid1b/WT transcripts from P10 mPFC. (n = 5 [WT], 5 [HT]).

(d and e) Volcano plots of DEGs (FDR < 0.05) in Arid1b/WT transcripts from P120 whole brain for the indicated comparisons. (n = 5 mice [WT-Veh], 5 [HT-Veh], 5 [WT-Mem], 5 [HT-Mem]).

(f and g) Volcano plots of DEGs (FDR < 0.05) in Arid1b/WT transcripts from P120

mPFC for the indicated comparisons. n = 4 mice [WT-Veh], 4 [HT-Veh], 4 [WT-Mem], 4 [HT-Mem]).

p values were obtained by Wald test and corrected for multiple comparisons using Benjamini & Hochberg with DESeq2 in the R package.

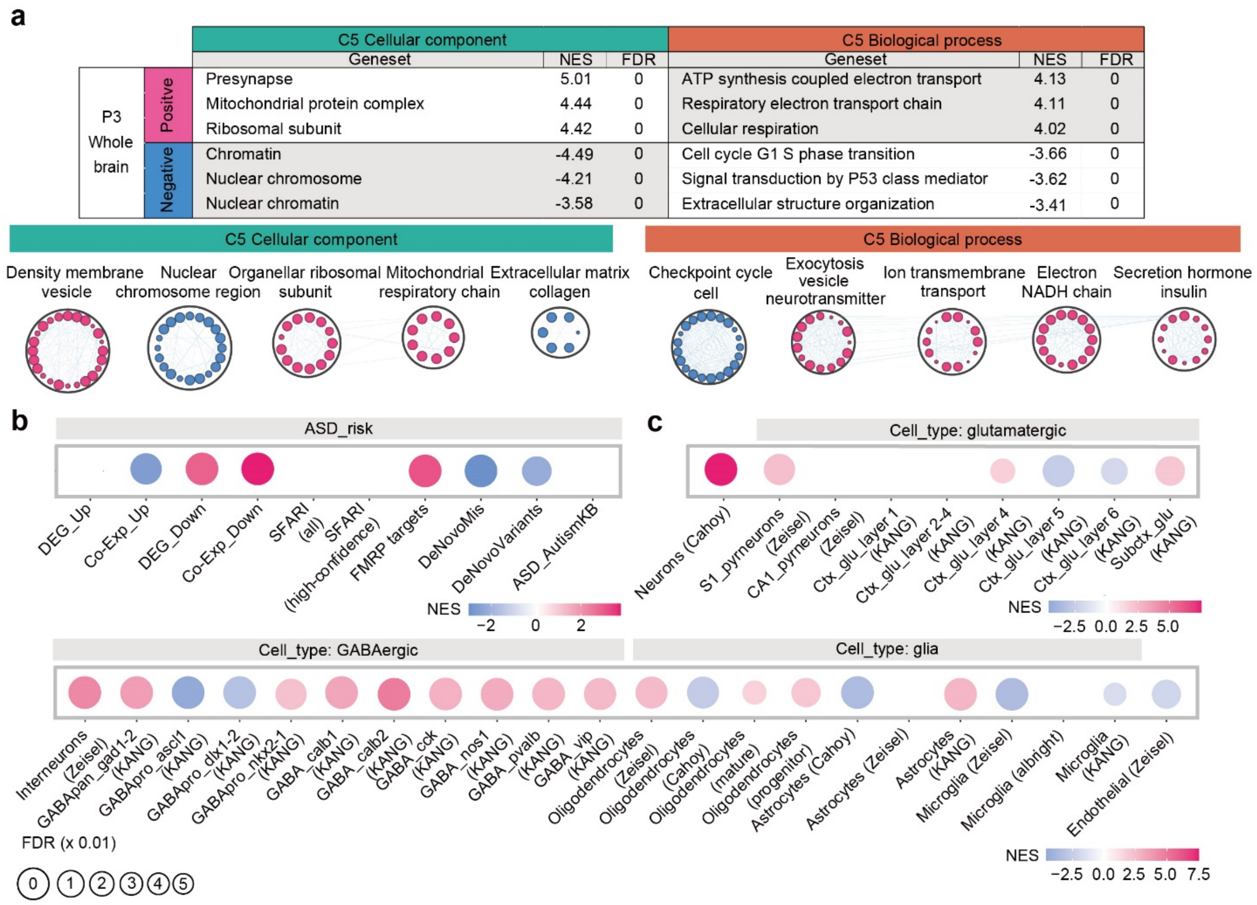

Supplementary Figure 7. Synapse- and ASD-related transcriptomic changes in the whole brain of *Arid1b*$^{+/-}$ mice at P3.

(a) List of top-five gene sets (ranked by p-value) positively or negatively enriched among Arid1b/WT transcripts from the whole-brain of P3 males, determined by GSEA. CC and BP, cellular component (CC) and biological process (BP) domains in the C5 gene-set database (top), and functional clustering of the enriched gene sets from the GSEA results using Cytoscape EnrichmentMap (bottom). Only the top-five clusters are shown to save space. NES, normalized enrichment score; FDR, false discovery rate. (n = 5 mice [WT and HT]).

(b) GSEA of Arid1b/WT P3 whole-brain transcripts for ASD-related/risk gene sets. (n = 5 mice [WT/HT]).

(c) GSEA of Arid1b/WT P3 whole-brain transcripts for cell-type–specific gene sets. (n = 5 mice [WT/HT]).

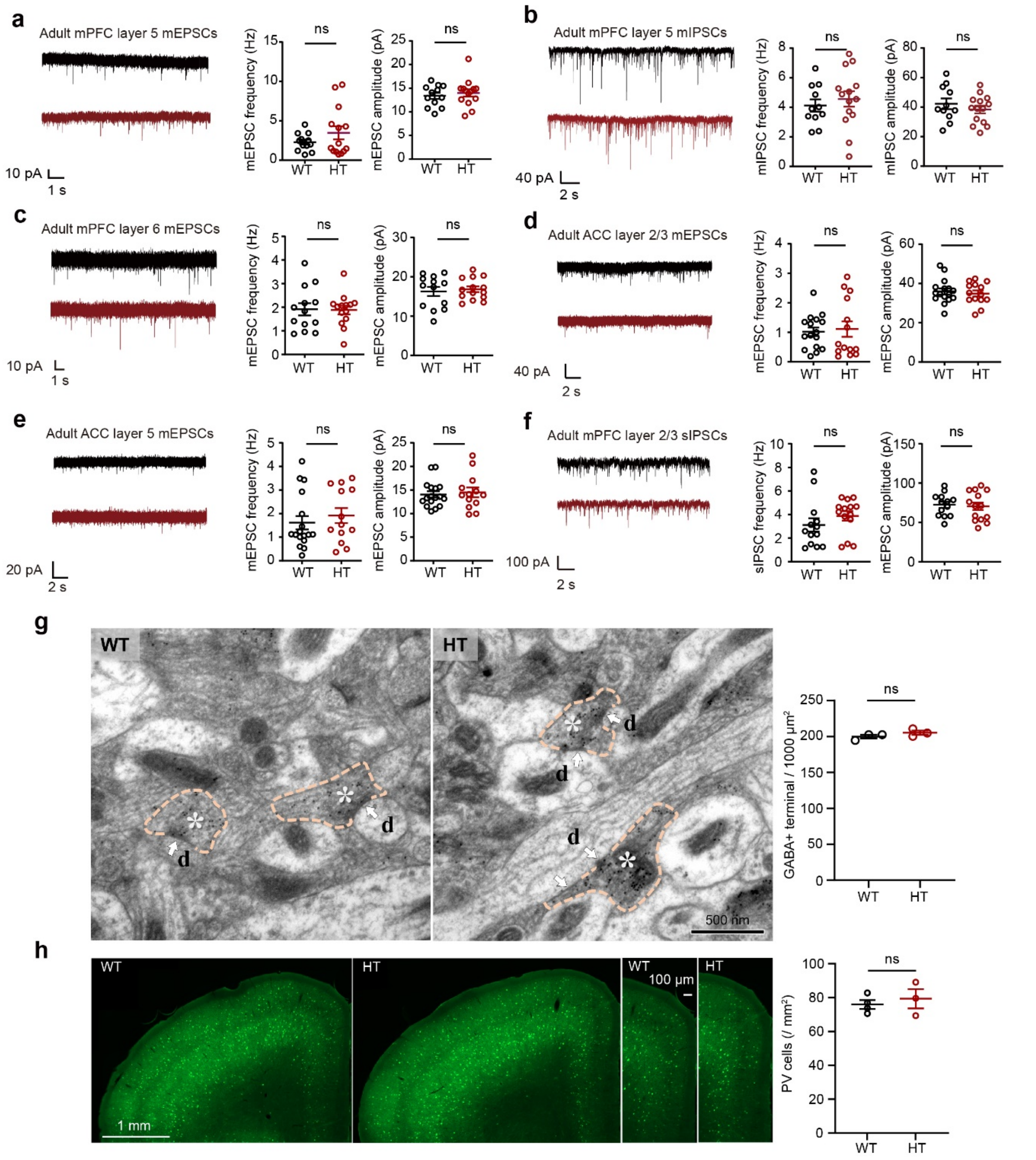

Supplementary Figure 8. Excitatory and inhibitory synaptic transmission, inhibitory synapse number, and Pv neuronal density in *Arid1b*$^{+/-}$ cortical pyramidal neurons.

(a) Normal mEPSC (miniature excitatory postsynaptic current) frequency and amplitude in layer 5 pyramidal neurons in the prelimbic region of the medial prefrontal cortex (mPFC) of Adult *Arid1b*$^{+/-}$ mice (~P42). (n = 12 neurons from 3 mice [WT] and 14, 3 [HT], two-tailed Mann-Whitney test [frequency], two-tailed Student's t-test [amplitude]).

(b) Normal mIPSC (miniature inhibitory postsynaptic current) frequency and

amplitude in layer 5 pyramidal neurons in the prelimbic region of the mPFC of Adult *Arid1b*$^{+/–}$ mice (~P56). (n = 11, 3 [WT] and 14, 3 [HT], two-tailed Student's t-test).

(c) Normal mEPSC frequency and amplitude in layer 6 pyramidal neurons in the prelimbic region of the mPFC of adult *Arid1b*$^{+/–}$ mice (~P56). (n = 13, 3 [WT] and 14, 3 [HT], two-tailed Student's t-test).

(d and e) Normal mEPSC frequency and amplitude in layer 2/3 and layer 5 pyramidal neurons in the anterior cingulate cortex (ACC) region in adult *Arid1b*$^{+/–}$ mice (~P56). (n = 16, 3 [WT] and 14, 3 [HT] for layer 2/3, n = 17, 3 [WT], 13, 3 [HT] for layer 5, two-tailed Mann-Whitney test [frequency], two-tailed Student's t-test [amplitude]).

(f) Normal sIPSC (spontaneous inhibitory postsynaptic current) frequency and amplitude in layer 2/3 pyramidal neurons in the prelimbic region of the mPFC of adult *Arid1b*$^{+/–}$ mice (~P56). (n = 13, 3 [WT] and 14, 3 [HT], two-tailed Mann-Whitney test [frequency], two-tailed Student's t-test [amplitude]).

(g) Normal numbers of inhibitory synapses, as determined by electron microscopic analysis of GABA-immunopositive axon terminals in the prelimbic region of the mPFC (layers 2/3) in WT and *Arid1b*$^{+/–}$ mice (~P56). (n = 3 mice [WT] and 3 [HT], two-tailed Student's t-test).

(h) Normal numbers of parvalbumin-positive GABA neurons in the prelimbic region of the mPFC (layers 2/3) in *Arid1b*$^{+/–}$ mice (~P56). (n = 3 mice [WT] and 3 [HT], two-tailed Student's t-test).

Graphical data are presented as means ± SEM (*p < 0.05, **p < 0.01, ***p < 0.001, ns, not significant). Source data are provided as a Source Data file.

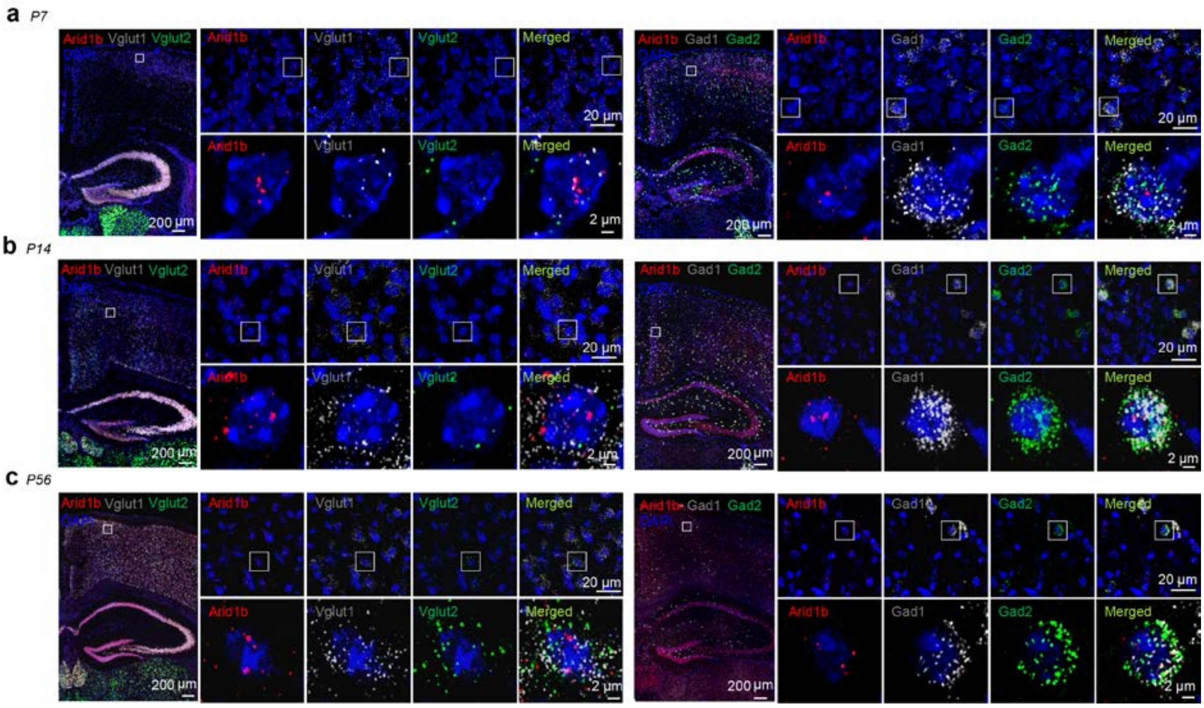

Supplementary Figure 9. Detection of *Arid1b* mRNA in both glutamatergic and GABAergic neurons in cortical areas.

(a–c) *Arid1b* mRNAs detected in glutamatergic and GABAergic neurons in the retrosplenial cortex of WT mice at P7, P14, and P56, detected by FISH analysis of coronal slices. Vglut1/2 and Gad1/2 were used as markers of glutamatergic and GABAergic neurons, respectively, and nuclei were stained with DAPI. Single-cell glutamatergic and GABAergic neurons are enlarged to show *Arid1b* mRNA colocalization with these neurons. Similar results were obtained from two independent experiments. P, postnatal. Scale bars, 200, 20, and 2 µm.

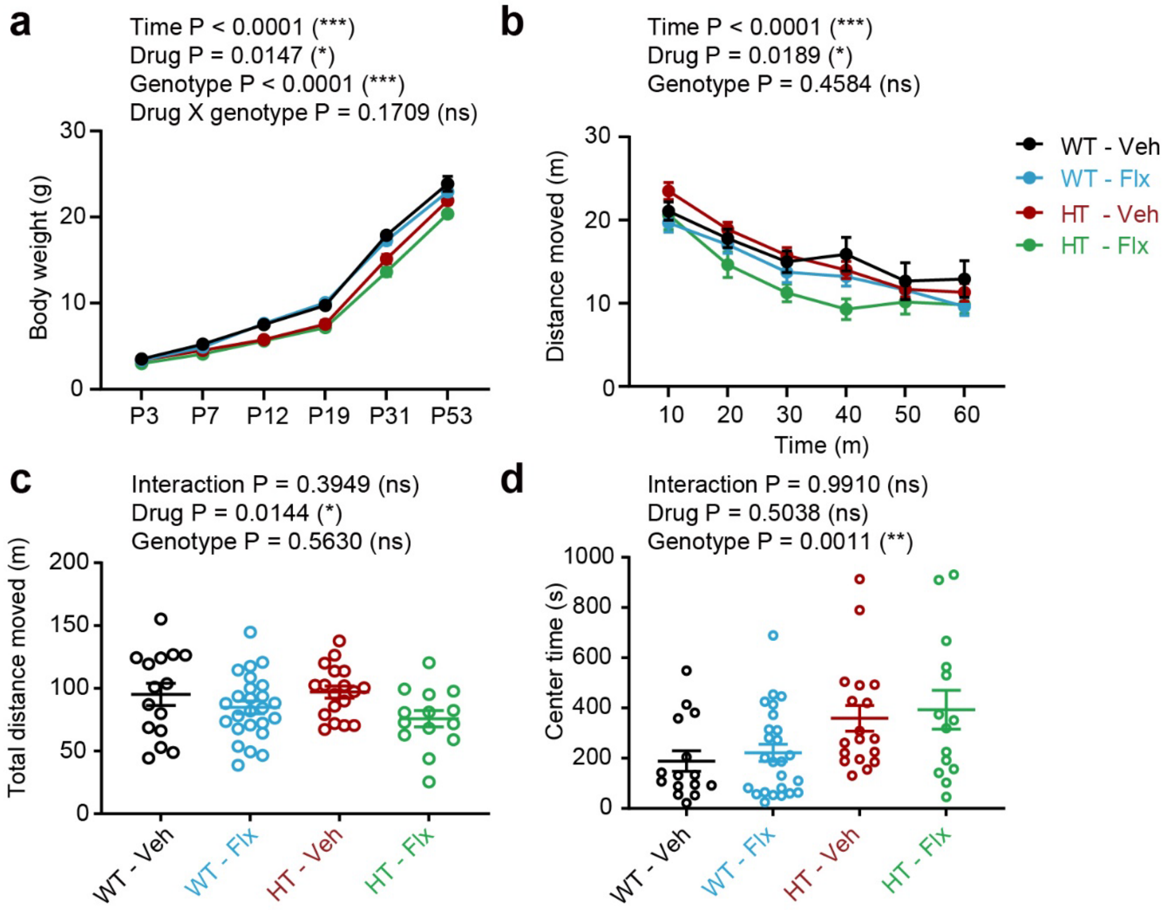

Supplementary Figure 10. Early, chronic fluoxetine treatment moderately decreases body weight and locomotor activity without affecting anxiety-like behavior in adult *Arid1b*$^{+/-}$ mice.

(a) Early, chronic (P3–21) fluoxetine treatment moderately decreases body weight in *Arid1b*$^{+/-}$ and WT mice. (n = 30 mice [WT-Veh], 42 [WT-Flx], 18 [HT-Veh], 30 [HT-Flx], three-way ANOVA, [Interaction: p = 0.4287]).

(b–d) Early, chronic fluoxetine treatment moderately decreases locomotor activity in adult (P60-90) *Arid1b*$^{+/-}$ and WT mice in the open-field test (100 lux), as shown by distance moved and total distance moved. Note that the time spent in the center region of the open-field arena, a measure of anxiety-like behavior, was not affected by early, chronic fluoxetine treatment in *Arid1b*$^{+/-}$ or WT mice. (n = 15 [WT-Veh], 25 [WT-Flx], 18 [HT-Flx], 12 [HT-Flx], three-way ANOVA [Distance moved], and two-way ANOVA [Total distance moved, and Center time], [Distance moved, interaction: p = 0.2886], [Total distance moved, interaction: p = 0.3949], [Center time, interaction: p = 0.9910]).

Graphical data are presented as means ± SEM (*p < 0.05, **p < 0.01, ***p < 0.001, ns, not significant). Source data are provided as a Source Data file.

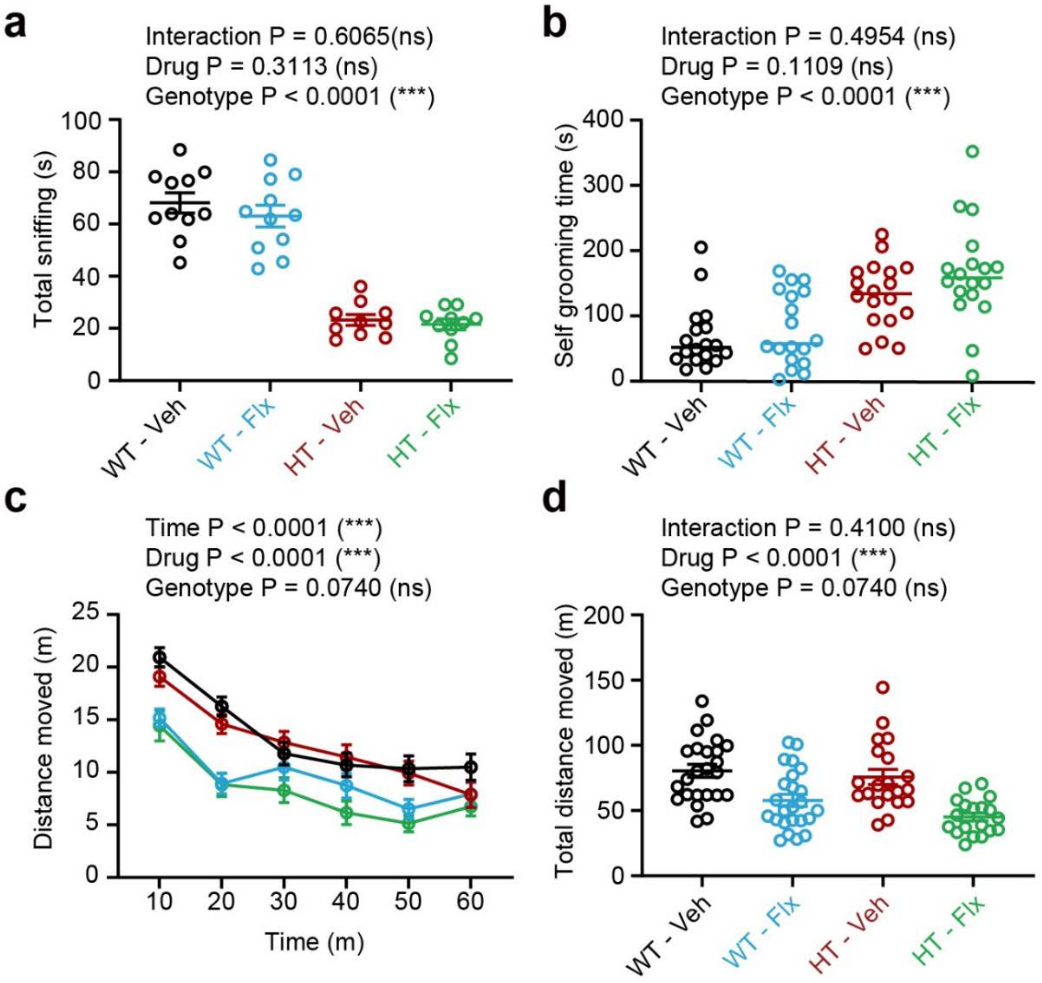

Supplementary Figure 11. Chronic fluoxetine treatment in adult *Arid1b*$^{+/-}$ mice does not rescue social interaction or self-grooming.

(a) Chronic fluoxetine treatment in adult *Arid1b*$^{+/-}$ mice (P100–200) does not rescue social defects, measured by the direct/dyadic social-interaction test. (n = 11 mouse pairs [WT-Veh], 11 [WT-Flx], 10 [HT-Veh], 10 [HT-Flx], two-way ANOVA, [Interaction: p = 0.6065]).

(b) Adult-stage, chronic fluoxetine (P100–200) does not rescue excessive self-grooming in adult *Arid1b*$^{+/-}$ mice (as shown by time spent self-grooming) without affecting grooming in WT mice. (n = 18 mice [WT-Veh], 18 [WT-Flx], 18 [HT-Veh], 18 [HT-Flx], two-way ANOVA, [Interaction: p = 0.4954]).

(c and d) Adult-stage, chronic fluoxetine (P100–200) moderately decreases locomotor activity in *Arid1b*$^{+/-}$ and WT mice in the open-field test. (n = 24 mice [WT-Veh], 24 [WT-Flx], 20 [HT-Veh], 19 [HT-Flx], three-way ANOVA [moved distance] [Interaction: p = 0.0130], and two-way ANOVA [total distance] [Interaction: p = 0.4100]).

Graphical data are presented as means ± SEM (*p < 0.05, **p < 0.01, ***p < 0.001, ns, not significant). Source data are provided as a Source Data file.

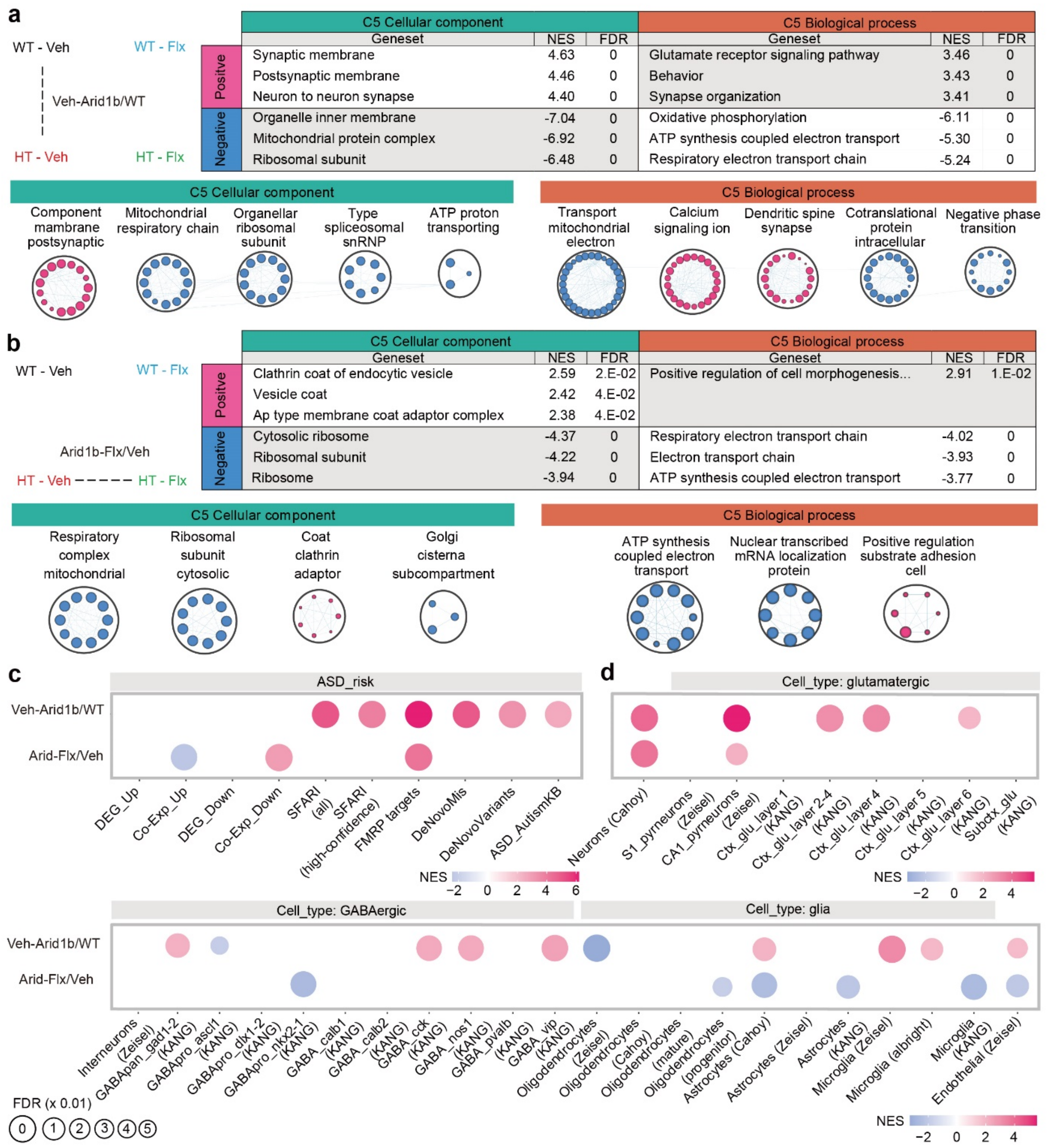

Supplementary Figure 12. Early, chronic fluoxetine treatment prevents aberrant transcriptomic changes in the mPFC of adult *Arid1b*$^{+/-}$ mice.

(a) Baseline differences in mPFC Arid1b/WT transcripts associated with biological functions in early vehicle-treated (P3–21) mice, measured in whole-brain lysates at P120 (Veh-HT/WT transcripts), as shown by the top-three enriched gene-set lists from GSEA using C5-CC and C5-BP gene sets and EnrichmentMap integration and visualization of enriched gene sets. NES, normalized enrichment score; FDR, false discovery rate. (n = 4 mice [WT-Veh], 4 [HT-Veh], 4 [WT-Mem], 4 [HT-Mem]).

(b) Transcriptomic changes associated with biological functions induced by early,

chronic fluoxetine treatment (P3–21) in the mPFC of adult *Arid1b*$^{+/-}$ mice, measured at P120 (Arid1b-Flx/Veh transcripts), as shown by the top-three enriched gene-set lists and EnrichmentMap integration/visualization. (n = 4 mice [WT-Veh], 4 [HT-Veh], 4 [WT-Mem], 4 [HT-Mem]).

(c) ASD-related and cell-type–specific transcriptomic changes in the mPFC in early vehicle-treated *Arid1b*$^{+/-}$ and WT mice (Veh-Arid1b/WT transcripts) and early fluoxetine/vehicle-treated *Arid1b*$^{+/-}$ mice (Arid1b-Flx/Veh transcripts), as shown by enrichment patterns for ASD-related/risk gene sets and cell-type–specific gene sets. (n = 4 mice [WT-Veh], 4 [HT-Veh], 4 [WT-Mem], 4 [HT-Mem]).

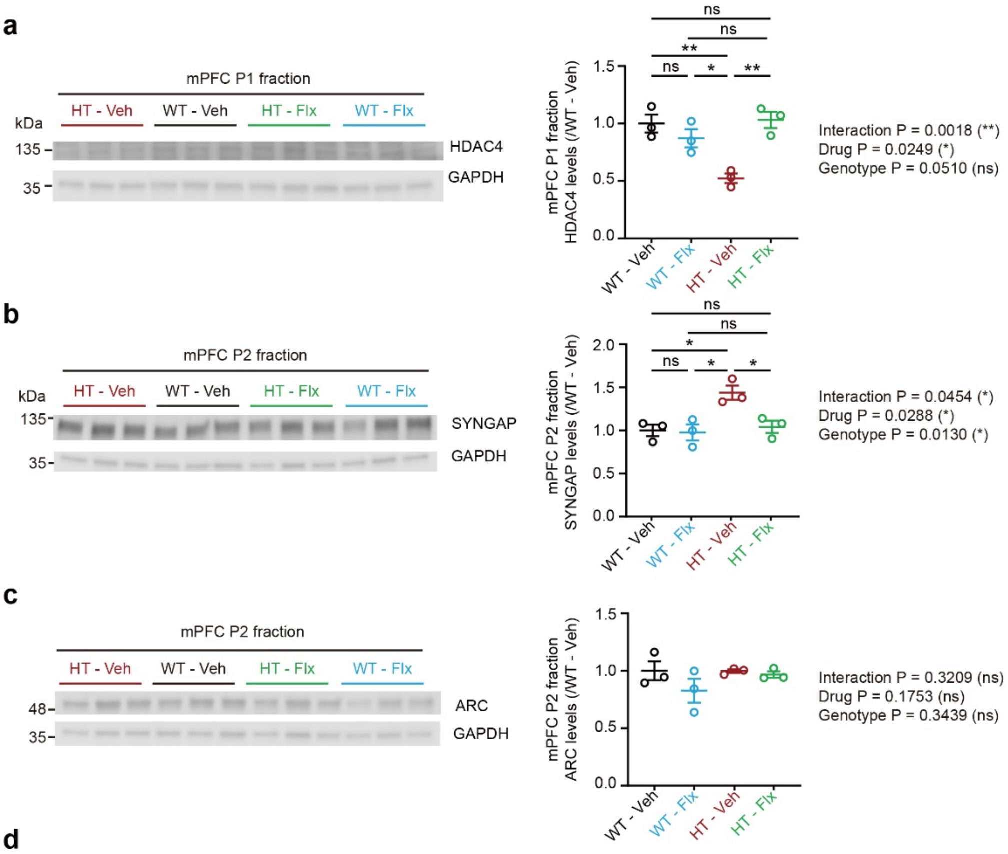

d

WT - Veh WT - Flx

Veh-Arid1b/WT

HT - Veh HT - Flx

Whole brain

Name	Fold change	FDR q-value
HDAC4	-1.02	9.75E-01
SYNGAP1	1.12	9.54E-01
ARC	-1.11	9.54E-01

mPFC

Name	Fold change	FDR q-value
HDAC4	-1.00	1.00E+00
SYNGAP1	1.05	9.98E-01
ARC	1.00	1.00E+00

WT - Veh WT - Flx

Arid1b-Flx/Veh

HT - Veh ------ HT - Flx

Whole brain

Name	Fold change	FDR q-value
HDAC4	1.32	9.53E-02
SYNGAP1	1.23	2.18E-01
ARC	1.23	4.49E-01

mPFC

Name	Fold change	FDR q-value
HDAC4	1.13	1.00E+00
SYNGAP1	1.01	1.00E+00
ARC	1.25	1.00E+00

Supplementary Figure 13. Early, chronic fluoxetine treatment normalizes HDAC4 and SynGAP1 levels in adult *Arid1b*$^{+/-}$ mice.

(a) Nuclear (P1 fraction) levels of HDAC4 protein in the mPFC of adult (P120) WT and *Arid1b*$^{+/-}$ mice treated early (P3–21) with vehicle or fluoxetine. (n = 3 [WT-Veh], 3 [WT-Flx], 3 [HT-Veh], and 3 [HT-Flx], two-way ANOVA with Tukey's test, [WT-Veh vs HT-Veh: p = 0.0054, WT-Veh vs WT-Flx: p = 0.5872, WT-Veh vs HT-Flx: p = 0.9871, HT-Veh vs WT-Flx: p = 0.0300, HT-Veh vs HT-Flx: p = 0.0037, WT-Flx vs HT-Flx: p = 0.4169]).

(b and c) Levels of SynGAP1 and Arc proteins in the crude synaptosomal (P2) mPFC fraction of adult (P120) WT and *Arid1b*$^{+/-}$ mice treated early with (P3–21)

vehicle or fluoxetine. (n = 3 [WT-Veh], 3 [WT-Flx], 3 [HT-Veh], and 3 [HT-Flx], two-way ANOVA with Tukey's test, [SynGAP, WT-Veh vs HT-Veh: $p = 0.0185$, WT-Veh vs WT-Flx: $p = 0.9966$, WT-Veh vs HT-Flx: $p = 0.9818$, HT-Veh vs WT-Flx: $p = 0.0140$, HT-Veh vs HT-Flx: $p = 0.0306$, WT-Flx vs HT-Flx: $p = 0.9370$]).

(d) Changes in the transcript levels for HDAC4, MEF2A, SynGAP1, and Arc in the whole brain and mPFC of adult (P120) WT and *Arid1b*$^{+/-}$ mice treated early with (P3–21) vehicle or fluoxetine. (n = 5 [WT-Veh], 5 [WT-Flx], 5 [HT-Veh], and 5 [HT-Flx] [Whole brain], and n = 4 [WT-Veh], 4 [WT-Flx], 4 [HT-Veh], and 4 [HT-Flx] [mPFC]).

Graphical data are presented as means ± SEM (*$p < 0.05$, **$p < 0.01$, ***$p < 0.001$, ns, not significant). Source data are provided as a Source Data file.

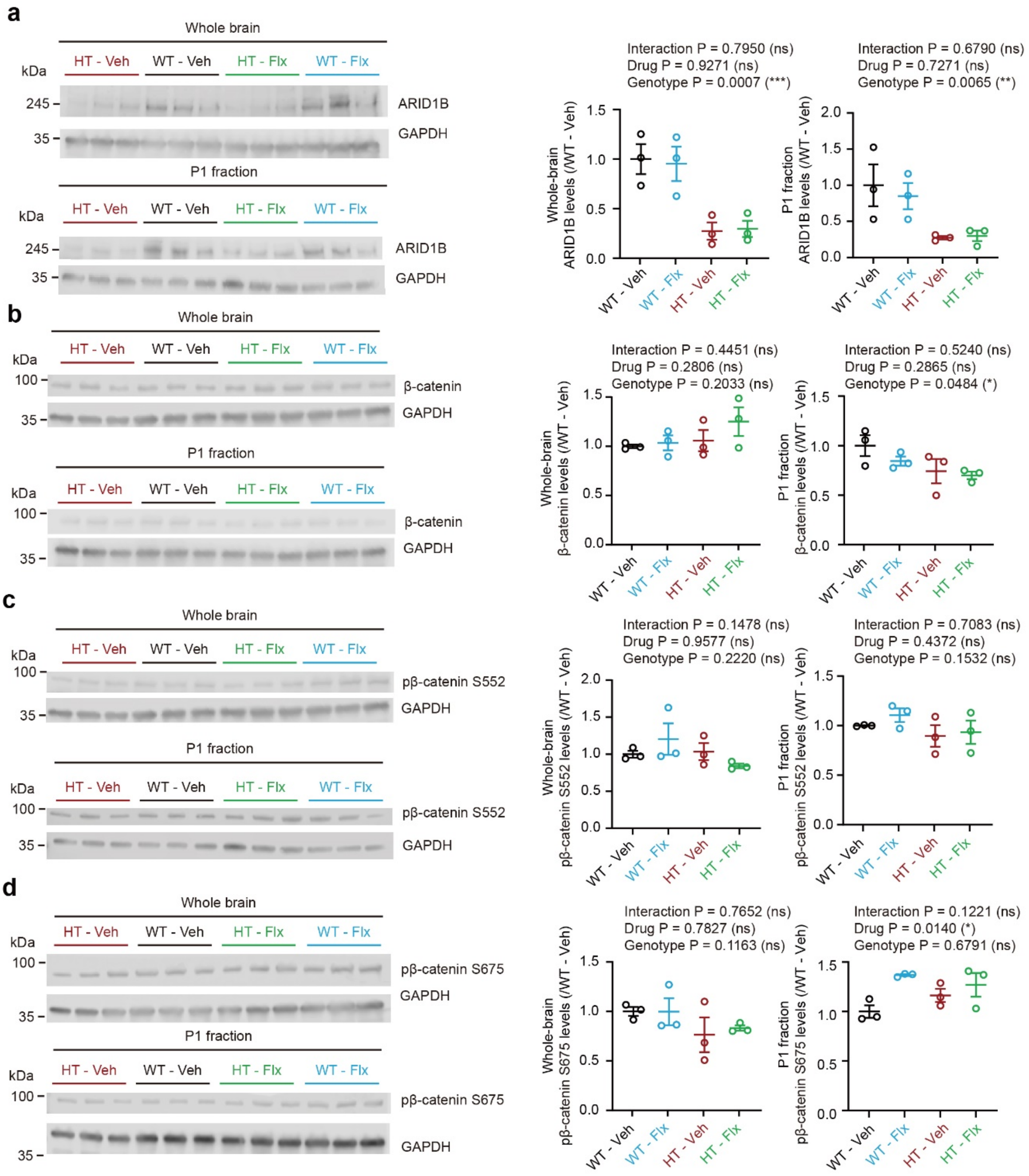

Supplementary Figure 14. Early, chronic fluoxetine treatment does not affect nuclear localization of β-catenin in *Arid1b*$^{+/-}$ mice.

(a) Whole brain and nuclear (P1 fraction) levels of ARID1B protein in adult (P120) WT and *Arid1b*$^{+/-}$ mice treated early (P3–21) with vehicle or fluoxetine. Note that early fluoxetine treatment does not affect total or P1 levels of ARID1B. (n = 3 [WT-Veh], 3 [WT-Flx], 3 [HT-Veh], and 3 [HT-Flx], two-way ANOVA).

(b) Whole brain and P1 levels of β-catenin protein in adult (P120) WT and *Arid1b*$^{+/-}$

mice treated early (P3–21) with vehicle or fluoxetine. (n = 3 [WT-Veh], 3 [WT-Flx], 3 [HT-Veh], and 3 [HT-Flx], two-way ANOVA).

(c) Whole brain and P1 levels of S552-phosphorylated β-catenin protein in adult (P120) WT and *Arid1b*$^{+/-}$ mice treated early (P3–21) with vehicle or fluoxetine. (n = 3 [WT-Veh], 3 [WT-Flx], 3 [HT-Veh], and 3 [HT-Flx], two-way ANOVA).

(d) Whole brain and P1 levels of S675-phosphorylated β-catenin protein in adult (P120) WT and *Arid1b*$^{+/-}$ mice treated early (P3–21) with vehicle or fluoxetine. (n = 3 [WT-Veh], 3 [WT-Flx], 3 [HT-Veh], and 3 [HT-Flx], two-way ANOVA).

Graphical data are presented as means ± SEM (*p < 0.05, **p < 0.01, ***p < 0.001, ns, not significant). Source data are provided as a Source Data file.

Supplementary Tables

Supplementary Table 1. Statistical details

Figure		Assay Performed	Parameter (Unit)	Comparison			N	Descriptive Statistics	Statistical Analysis					Normality test	
				Sex	Age	groups		Average ± SEM	Statistical Test		Exact P value, Significance			D'Agostino & Pearson normality test	Shapiro-Wilk normality test
1	b	Whole brain<br>western blot: ARID1B	Relative protein levels	male	P0	WT, HT, KO	3, 3, 3	Oridinary ONE-WAY ANOVA			P < 0.0001<br>F = 66.10				
								Dunnett's multiple comparison		WT vs HT	P = 0.0005, ***				
										WT vs KO	P < 0.0001, ***				
	d	Direct interaction test	Total sniffing time (s)	male	P60-P90	WT, HT	10 (20), 12 (24)	WT = 80.63 ± 11.32<br>HT = 25.41 ± 3.402	Students' t-test		P < 0.0001, ***	t=5.048 df=20	Two-tailed	P = 0.2883, ns<br>P = 0.4494, ns	P = 0.2052, ns<br>P = 0.0957, ns
			Nose to nose time (s)				10 (20), 12 (24)	WT = 16.09 ± 1.424<br>HT = 11.04 ± 1.166	Students' t-test		P = 0.0117, *	t=2.773 df=20	Two-tailed	P = 0.9375, ns<br>P = 0.0780, ns	P = 0.8333, ns<br>P = 0.1550, ns
			Nose to body time (s)				10 (20), 12 (24)	WT = 64.54 ± 10.89<br>HT = 14.36 ± 2.769	Welch's t test		P = 0.0012, **	t=4.464 df=10.17	Two-tailed	P = 0.2600, ns<br>P = 0.2888, ns	P = 0.0542, ns<br>P = 0.0704, ns
			Following time (s)				10 (20), 11 (22)	WT = 18.57 ± 3.836<br>HT = 10.51 ± 1.970	Students' t-test		P = 0.0697, ns	t=1.922, df=19	Two-tailed	P = 0.8000, ns<br>P = 0.1650, ns	P = 0.7890, ns<br>P = 0.0824, ns
	e	3-Chamber test	Preference index (S1 vs O)	male	P60-P90	WT, HT	17, 13	WT = 76.93 ± 4.939<br>HT = 71.88 ± 6.791	Student's t-test		P = 0.5420, ns	t=0.6174, df=28	Two-tailed	P = 0.2865, ns<br>P = 0.3661, ns	P = 0.0661, ns<br>P = 0.0938, ns
			Preference index (S1 vs S2)			WT, HT	17, 13	WT = 37.69 ± 15.5<br>HT = 35.59 ± 12.13	Mann-Whitney test		P = 0.6128, ns	U = 98	Two-tailed	P = 0.0785, ns<br>P = 0.3833, ns	P = 0.0097, **<br>P = 0.3736, ns
	f	Pup USV	Call number	Male	P3-11	WT, HT	14 [male-WT], 17 [female-WT],<br>17 [male-HT], 12 [female-HT]	2-WAY ANOVA			Interaction :: P = 0.0272, DF=4<br>Age :: P < 0.0001, DF = 4<br>Genotype :: P < 0.0001, DF = 1				
								Sidak's Multiple Copmarison	P3	WT vs HT	P = 0.0040, **				
									P5	WT vs HT	P = 0.0062, **				
									P7	WT vs HT	P < 0.0001, ***				
									P9	WT vs HT	P < 0.0001, ***				
									P11	WT vs HT	P = 0.0003, ***				
	g	Grooming	Time spent grooming (s)	male	P60-P90	WT, HT	23, 25	WT = 73.02 ± 12.2<br>HT = 144 ± 21.72	Mann-Whitney test		P = 0.0322, *	U = 184	Two-tailed	P = 0.2411, ns<br>P = 0.0825, ns	P = 0.0466, *<br>P = 0.0369, *
3	a	mPFC Layer II/III, mEPSC	Frequency (Hz)	male	P19~21	WT, HT	18(3), 18(3)	WT = 4.293 ± 0.3196<br>HT = 2.333 ± 0.2765	Students' t-test		P<0.0001, ***	t=4.637, df=34	Two-tailed	P = 0.9699, ns<br>P = 0.1119, ns	P = 0.9800, ns<br>P = 0.1109, ns
			Amplitude (pA)	male		WT, HT	18(3), 18(3)	WT = 24.66 ± 1.139<br>HT = 22.9 ± 0.9146	Mann-Whitney test		P = 0.1916, ns	U = 120	Two-tailed	P = 0.1649, ns<br>P = 0.0019, **	P = 0.0602, ns<br>P = 0.0019, **
	b	mPFC Layer II/III, mIPSC	Frequency (Hz)	male	P19~21	WT, HT	16(3), 14(3)	WT = 9.169 ± 0.6959<br>HT = 10.05 ± 1.026	Students' t-test		P = 0.4739, ns	t=0.7259, df=28	Two-tailed	P = 0.5009, ns<br>P = 0.3785, ns	P = 0.7014, ns<br>P = 0.0730, ns
			Amplitude (pA)	male		WT, HT	16(3), 14(3)	WT = 35.36 ± 2.294<br>HT = 33.14 ± 2.26	Mann-Whitney test		P = 0.7587, ns	U = 104	Two-tailed	P = 0.0052, **<br>P = 0.8655, ns	P = 0.0250, *<br>P = 0.8703, ns
	d	Quantitative measurement of PSD using electron microscopy	Number of PSD / 1000 μm2	male	P21	WT, HT	3 (96 EM), 3 (96 EM)	WT = 308.6 ± 4.398<br>HT = 230.7 ± 2.669	Students' t-test		P = 0.0001, ***	t=15.13, df=4	Two-tailed		P = 0.5819, ns<br>P = 0.7259, ns
	e	Quantitative measurement of PSD using electron microscopy	Length of PSD (μm)	male	P21	WT, HT	3 (264, 288, 277), 3 (206, 198, 201)	WT = 0.1877 ± 0.002028<br>HT = 0.1863 ± 0.001453	Students' t-test		P = 0.6213, ns	t=0.5345, df=4	Two-tailed		P = 0.8428, ns<br>P = 0.7804, ns
	f	Quantitative measurement of PSD using electron microscopy	Thickness of PSD (μm)	male	P21	WT, HT	3 (264, 288, 277), 3 (206, 198, 201)	WT = 0.014 ± 0.0005774<br>HT = 0.01433 ± 0.0006667	Mann-Whitney test		P = 0.8137, ns	U = 3.5	Two-tailed		P > 0.9999, ns<br>P < 0.0001, ****
	g	Quantitative measurement of PSD using electron microscopy	Perforated synapse (%)	male	P21	WT, HT	3 (96 EM), 3 (96 EM)	WT = 7.293 ± 0.8396<br>HT = 7.197 ± 0.6987	Students' t-test		P = 0.9337, ns	t=0.0885, df=4	Two-tailed		P = 0.4305, ns<br>P = 0.9636, ns
	h	mPFC layer II/III, mEPSC	Frequency (Hz)	male	P42	WT, HT	13 (3), 21(4)	WT = 4.466 ± 0.3976<br>HT = 2.865 ± 0.2389	Mann-Whitney test		P = 0.0053, **	U = 59.5	Two-tailed	P = 0.0090, **<br>P = 0.1105, ns	P = 0.0049, **<br>P = 0.1278, ns
			Amplitude (pA)	male		WT, HT	13 (3), 21(4)	WT = 13.61 ± 0.3053<br>HT = 12.99 ± 0.3463	Mann-Whitney test		P = 0.2308, n.s	t=1.222, df=32	Two-tailed	P = 0.2971, ns<br>P = 0.5669, ns	P = 0.0937, ns<br>P = 0.4428, ns
	i	mPFC layer II/III, mIPSC	Frequency (Hz)	male	P56	WT, HT	14 (3), 14 (3)	WT = 5.379 ± 0.3795<br>HT = 5.161 ± 0.282	Students' t-test		P = 0.6494, ns	t=0.4599 df=26	Two-tailed	P = 0.8851, ns<br>P = 0.6163, ns	P = 0.7321, ns<br>P = 0.5049, ns
			Amplitude (pA)	male		WT, HT	14 (3), 14 (3)	WT = 56.12 ± 2.464<br>HT = 58.92 ± 2.43	Students' t-test		P = 0.4249, ns	t=0.8107 df=26	Two-tailed	P = 0.8007, ns<br>P = 0.8959, ns	P = 0.9923, ns<br>P = 0.4554, ns
	j	mPFC layer II/III, PPR	% Facilitation	male	P42	WT, HT	8(3), 6(3)	2-WAY ANOVA			Interaction :: P = 0.7241, ns, DF = 5<br>Time :: P = 0.0022, **, DF = 5<br>Genotype :: P = 0.7424, ns, DF = 1				
	k	mPFC layer II/III, resting membrane potential	RMP (mV)	male	P56~P70	WT, HT	14 (5), 15 (5)	WT = -74.79 ± 2.259<br>HT = -74.33 ± 2.018	Students' t-test		P = 0.8821, ns	t=0.1498, df=27	Two-tailed	P = 0.6496, ns<br>P = 0.2763, ns	P = 0.5701, ns<br>P = 0.6464, ns
	l	mPFC Layer II/III, Threshold	Threshold (mV)	male	P56~P70	WT, HT	13 (5), 15 (5)	WT = -28.85 ± 2.712<br>HT = -29.07 ± 2.345	Students' t-test		P = 0.9512, ns	t=0.06184, df=26	Two-tailed	P = 0.4237, ns<br>P = 0.6977, ns	P = 0.1759, ns<br>P = 0.9330, ns
	m	mPFC layer II/III, Firing Rate	# of Action Potential	male	P56~P70	WT, HT	14 (5), 15 (5)	2-WAY ANOVA			Interaction :: P = 0.9940, ns, DF = 10<br>Time :: P < 0.0001, ****, DF = 10<br>Genotype :: P = 0.7711, ns, DF = 1				
	n	mPFC layer II/III, Input resistance	Voltage (mV)	male	P56~P70	WT, HT	14 (5), 15 (5)	2-WAY ANOVA			Interaction :: P = 0.9824, ns, DF = 2<br>Time :: P < 0.0001, ****, DF = 2<br>Genotype :: P = 0.9489, ns, DF = 1				
4	d	Direct interaction test	Time sniffing time (s)	Male	P60-P90	WT (VEH), WT (FLX), HT (VEH), HT (FLX)	10 (20), 14 (28), 12 (24), 10 (20)	2-WAY ANOVA			Interaction :: P = 0.0425, DF=1<br>Drug :: P = 0.0157, DF = 1<br>Genotype :: P < 0.0001, DF = 1				
								Tukey's Multiple Copmarison		VEH:WT vs. VEH:HT	P < 0.0001, ***				
										VEH:WT vs. FLX:WT	P = 0.9899, ns				
										VEH:WT vs. FLX:HT	P = 0.0058, **				
										VEH:HT vs. FLX:WT	P < 0.0001, ***				
										VEH:HT vs. FLX:HT	P = 0.0132, *				
										FLX:WT vs. FLX:HT	P = 0.0010, **				
	c	Grooming	Time spent in grooming (s)	Male	P60-P90	WT (VEH), WT (FLX), HT (VEH), HT (FLX)	13, 26, 18, 12	2-WAY ANOVA			Interaction :: P = 0.0008, DF=1<br>Drug :: P = 0.0004, DF = 1<br>Genotype :: P = 0.2813, DF = 1				
								Tukey's Multiple Copmarison		VEH:WT vs. VEH:HT	P = 0.0119, *				
										VEH:WT vs. FLX:WT	P = 0.9974, ns				
										VEH:WT vs. FLX:HT	P = 0.3400, ns				
										VEH:HT vs. FLX:WT	P = 0.0010, ***				
										VEH:HT vs. FLX:HT	P <0.0001, ***				
										FLX:WT vs. FLX:HT	P = 0.3099, ns				
5	b	mPFC Prelimbic cortex Layer II/III, mEPSC	Frequency (Hz)	Male	P100~P120	WT (VEH), WT (FLX), HT (VEH), HT (FLX)	19 (4), 14 (3), 16 (3), 16 (3)	2-WAY ANOVA			Interaction :: P = 0.0431, DF=1<br>Drug :: P = 0.0003, DF = 1<br>Genotype :: P = 0.1215, DF = 1				
								Tukey's Multiple Copmarison		VEH:WT vs. VEH:HT	P = 0.0462, *				
										VEH:WT vs. FLX:WT	P = 0.5802, ns				
										VEH:WT vs. FLX:HT	P = 0.3373, ns				
										VEH:HT vs. FLX:WT	P = 0.0025, **				
										VEH:HT vs. FLX:HT	P = 0.0005, ***				
										FLX:WT vs. FLX:HT	P = 0.9866, ns				
			Amplitude (pA)	Male	P100~P120	WT (VEH), WT (FLX), HT (VEH), HT (FLX)	19 (4), 14 (3), 16 (3), 17 (3)	2-WAY ANOVA			Interaction :: P = 0.3810, DF=1<br>Drug :: P = 0.0165, DF = 1<br>Genotype :: P = 0.4256, DF = 1				
								Sidak's Multiple Copmarison		VEH:WT vs. VEH:HT	P = 0.5184				
										VEH:WT vs. FLX:WT	P = 0.0640				
										VEH:WT vs. FLX:HT	P = 0.0594				
	c	mPFC Prelimbic cortex Laeyr II/III, PPR	% Facilitation	male	P100~P120	WT (VEH), WT (FLX), HT (VEH), HT (FLX)	17 (3), 11 (3), 16 (3), 12 (3)	3-WAY ANOVA			Intertime :: P < 0.0001, ***, DF = 5<br>Drug :: P = 0.9705, ns, DF = 1<br>Genotype :: P = 0.6799, ns, DF = 1<br>Intertime X Drug :: P = 0.6320, ns, DF = 5<br>Intertime X Genotype :: P = 0.2272, ns, DF = 5<br>Drug X Genotype :: P = 0.0687, ns, DF = 1<br>Intertime X Drug X Genotype :: P = 0.5047, ns, DF = 5				
	e	Quantitative measurement of PSD using electron microscopy	Number of PSD / 1000 μm2	male	P120	WT (VEH), WT (FLX), HT (VEH), HT (FLX)	5 (32 EM), 5 (32 EM), 5 (32 EM), 5 (32 EM)	2-WAY ANOVA			Interaction :: P < 0.0001, DF=1<br>Drug :: P< 0.0001, DF = 1<br>Genotype :: P < 0.0001, DF = 1				
								Tukey's Multiple Copmarison		VEH:WT vs. VEH:HT	P < 0.0001, ***				
										VEH:WT vs. FLX:WT	P = 0.6609, ns				
										VEH:WT vs. FLX:HT	P = 0.8977, ns				
										VEH:HT vs. FLX:WT	P < 0.0001, ***				
										VEH:HT vs. FLX:HT	P <0.0001, ***				
										FLX:WT vs. FLX:HT	P = 0.9661, ns				
	f	Quantitative measurement of PSD using electron microscopy	Length of PSD (μm)	male	P120	WT (VEH), WT (FLX), HT (VEH), HT (FLX)	5 (286, 297, 296, 303, 305), 5 (307, 311, 294, 294, 306),<br>5 (213, 202, 207, 204, 200),<br>5 (300, 294, 296, 301, 311)	2-WAY ANOVA			Interaction :: P = 0.4666, DF=1<br>Drug :: P = 0.8313, DF = 1<br>Genotype :: P = 0.7585, DF = 1				
	g	Quantitative measurement of PSD using electron microscopy	Thickness of PSD (μm)	male	P120	WT (VEH), WT (FLX), HT (VEH), HT (FLX)	5 (286, 297, 296, 303, 305), 5 (307, 311, 294, 294, 306),<br>5 (213, 202, 207, 204, 200),	2-WAY ANOVA			Interaction :: P = 0.6938, DF=1<br>Drug :: P = 0.5136, DF = 1<br>Genotype :: P = 0.8954, DF = 1				
	h	Quantitative measurement of PSD using electron microscopy	Perforated synapse (%)	male	P120	WT (VEH), WT (FLX), HT (VEH), HT (FLX)	5 (32 EM), 5 (32 EM), 5 (32 EM), 5 (32 EM)	2-WAY ANOVA			Interaction :: P = 0.9401, DF=1<br>Drug :: P = 0.4663, DF = 1<br>Genotype :: P = 0.2823, DF = 1				
7	c	Western blot: HDAC4	Whole brain<br>relative expression levels	Male	P120	WT (VEH), WT (FLX), HT (VEH), HT (FLX)	3, 3, 3, 3	2-WAY ANOVA			Interaction :: P = 0.031, DF=1<br>Drug :: P = 0.0563, DF = 1<br>Genotype :: P = 0.0379, DF = 1				
								Tukey's Multiple Copmarison		VEH:WT vs. VEH:HT	P = 0.0285, *				
										VEH:WT vs. FLX:WT	P = 0.9925, ns				
										VEH:WT vs. FLX:HT	P = 0.9978, ns				
										VEH:HT vs. FLX:WT	P = 0.0416, *				
										VEH:HT vs. FLX:HT	P = 0.0366, *				
										FLX:WT vs. FLX:HT	P = 0.9997, ns				
			P1 fraction<br>relative expression levels	Male	P120	WT (VEH), WT (FLX), HT (VEH), HT (FLX)	3, 3, 3, 3	2-WAY ANOVA			Interaction :: P = 0.2016, DF=1<br>Drug :: P = 0.0066, DF = 1<br>Genotype :: P = 0.1099, DF = 1				
								WT(VEH) = 1 ± 0.08563<br>HT(VEH) = 0.6412 ± 0.03251	Students' t-test	VEH:WT vs. VEH:HT	P = 0.0173, *	t=3.917, df=4	Two-tailed		
								WT(VEH) = 1 ± 0.08563<br>WT(FLX) = 1.253 ± 0.096	Students' t-test	VEH:WT vs. FLX:WT	P = 0.1209, ns	t=1.965, df=4	Two-tailed		
								WT(VEH) = 1 ± 0.08563<br>HT(FLX) = 1.207 ± 0.1817	Students' t-test	VEH:WT vs. FLX:HT	P = 0.3610, ns	t=1.031, df=4	Two-tailed		
								HT(VEH) = 0.6412 ± 0.03251<br>WT(FLX) = 1.253 ± 0.096	Students' t-test	VEH:HT vs. FLX:WT	P = 0.0038, **	t=6.034, df=4	Two-tailed		
								HT(VEH) = 0.6412 ± 0.03251<br>HT(FLX) = 1.207 ± 0.1817	Students' t-test	VEH:HT vs. FLX:HT	P = 0.0375, *	t=3.065, df=4	Two-tailed		
								WT(FLX) = 1.253 ± 0.096<br>HT(FLX) = 1.207 ± 0.1817	Students' t-test	FLX:WT vs. FLX:HT	P = 0.8347, ns	t=0.2227, df=4	Two-tailed		
	d	Western blot: MEF2A	Whole brain<br>relative expression levels	Male	P120	WT (VEH), WT (FLX), HT (VEH), HT (FLX)	3, 3, 3, 3	2-WAY ANOVA			Interaction :: P = 0.9835, DF=1<br>Drug :: P = 0.5574, DF = 1<br>Genotype :: P = 0.2893, DF = 1				
		Western blot: phospho-MEF2A S408	Whole brain<br>relative expression levels	Male	P120	WT (VEH), WT (FLX), HT (VEH), HT (FLX)	3, 3, 3, 3	2-WAY ANOVA			Interaction :: P = 0.1786, DF=1<br>Drug :: P = 0.0181, DF = 1<br>Genotype :: P = 0.0237, DF = 1				
								WT(VEH) = 1 ± 0.1704<br>HT(VEH) = 0.3529 ± 0.02593	Students' t-test	VEH:WT vs. VEH:HT	P = 0.0199, *	t=3.754, df=4	Two-tailed		
								WT(VEH) = 1 ± 0.1704<br>WT(FLX) = 0.9593 ± 0.1264	Students' t-test	VEH:WT vs. FLX:WT	P = 0.8571, ns	t=0.1920, df=4	Two-tailed		
								WT(VEH) = 1 ± 0.1704<br>HT(FLX) = 1.026 ± 0.1062	Students' t-test	VEH:WT vs. FLX:HT	P = 0.9018, ns	t=0.1315, df=4	Two-tailed		
								HT(VEH) = 0.3529 ± 0.02593<br>WT(FLX) = 0.9593 ± 0.1264	Students' t-test	VEH:HT vs. FLX:WT	P = 0.0093, **	t=4.700, df=4	Two-tailed		
								HT(VEH) = 0.3529 ± 0.02593<br>HT(FLX) = 1.026 ± 0.1062	Students' t-test	VEH:HT vs. FLX:HT	P = 0.0035, **	t=6.159, df=4	Two-tailed		
								WT(FLX) = 0.9593 ± 0.1264<br>HT(FLX) = 1.026 ± 0.1062	Students' t-test	FLX:WT vs. FLX:HT	P = 0.7051, ns	t=0.4067, df=4	Two-tailed		
	e	Western blot: SynGAP	Whole brain<br>relative expression levels	Male	P120	WT (VEH), WT (FLX), HT (VEH), HT (FLX)	3, 3, 3, 3	2-WAY ANOVA			Interaction :: P = 0.0282, DF=1<br>Drug :: P = 0.0301, DF = 1<br>Genotype :: P = 0.1345, DF = 1				
								Tukey's Multiple Copmarison		VEH:WT vs. VEH:HT	P = 0.0605, ns				
										VEH:WT vs. FLX:WT	P > 0.9999, ns				
										VEH:WT vs. FLX:HT	P = 0.9007, ns				
										VEH:HT vs. FLX:WT	P = 0.0631, ns				
										VEH:HT vs. FLX:HT	P = 0.0233, *				
										FLX:WT vs. FLX:HT	P = 0.8893, ns				
		Western blot: Arc	Whole brain<br>relative expression levels	Male	P120	WT (VEH), WT (FLX), HT (VEH), HT (FLX)	3, 3, 3, 3	2-WAY ANOVA			Interaction :: P = 0.0050, DF=1<br>Drug :: P = 0.0374, DF = 1<br>Genotype :: P = 0.0604, DF = 1				
								Tukey's Multiple Copmarison		VEH:WT vs. VEH:HT	P = 0.0118, *				
										VEH:WT vs. FLX:WT	P = 0.7801, ns				
										VEH:WT vs. FLX:HT	P = 0.9961, ns				
										VEH:HT vs. FLX:WT	P = 0.0432, *				
										VEH:HT vs. FLX:HT	P = 0.0089, *				
										FLX:WT vs. FLX:HT	P = 0.6623, ns				
8	a	LC-MS for 5-HT	Concentration (ng/g)	male	P10	WT (VEH), WT (FLX), HT (VEH), HT (FLX)	6, 6, 3, 6	2-WAY ANOVA			Interaction :: P = 0.9230, ns, DF = 1<br>Drug :: P = 0.5395, ns, DF = 1<br>Genotype :: P = 0.2409, ns, DF = 1				
	b	Arid1b HT Forced Swim Test	Immobilized time (s)	Male	P80	WT, HT	15, 13	WT = 62.54 ± 6.639<br>HT = 73.27 ± 9.844	Student's t-test		P = 0.3632, ns	t=0.9256 df=26	Two-tailed	P = 0.7966, ns<br>P = 0.1476, ns	P = 0.6087, ns<br>P = 0.5240, ns
	e	Arid1b SERT cHT Direct interaction with C3H	Total sniffing time (s)	Male	P80	WT, cHT	14, 9	WT = 48.49 ± 7.172<br>cHT = 45.22 ± 4.268	Mann-Whitney test		P = 0.5571, ns	U = 53	Two-tailed	P = 0.3838, ns<br>P = 0.0116, *	P = 0.1032, ns<br>P = 0.0519, ns
	f	Arid1b SERT cHT Grooming	Time spent in grooming (s)	Male	P80	WT, cHT	11, 11	WT = 66.07 ± 11.12<br>cHT = 50.06 ± 10.79	Student's t-test		P = 0.3136, ns	t=1.034 df=20	Two-tailed	P = 0.2107, ns<br>P = 0.3109, ns	P = 0.0745, ns<br>P = 0.0603, ns
S1	d	Body Weight	g	Male, Female	P3-49	WT, HT	15 [male-WT], 17 [female-WT],<br>17 [male-HT], 12 [female-HT]	Mixed effects analysis			Time :: P < 0.0001, F (1.536, 88.57) = 1558<br>Genotype :: P < 0.0001, F (1, 59) = 21.18<br>Time X Genotype :: P < 0.0001, F (6, 346) = 18.00				
								Sidak's Multiple Copmarison	P3	WT vs HT	P = 0.2649, ns				
									P5	WT vs HT	P = 0.3043, ns				
									P7	WT vs HT	P = 0.1350, ns				
									P9	WT vs HT	P = 0.0154, *				
									P11	WT vs HT	P = 0.0014, **				
									P21	WT vs HT	P < 0.0001, ***				
									P49	WT vs HT	P = 0.0006, ***				
S2	b	Brain subregion size measure	Cortex / Half brain (ratio)	male	P42	WT, HT	4, 4	WT = 0.1084 ± 0.002011<br>HT = 0.1131 ± 0.003723	Students' t-test		P = 0.3090, ns	t=1.111, df=6	Two-tailed		P = 0.2263, ns<br>P = 0.522, ns
			Hippocampus / Half brain (ratio)	male	P42	WT, HT	4, 4	WT = 0.1774 ± 0.008981<br>HT = 0.1711 ± 0.002213	Student's t-test		P = 0.5252, ns	t=0.6744, df=6	Two-tailed		P = 0.6086, ns<br>P = 0.3853, ns
			Thalamus / Half brain (ratio)	male	P42	WT, HT	4, 4	WT = 0.343 ± 0.003743<br>HT = 0.3518 ± 0.004858	Student's t-test		P = 0.2053, ns	t=1.42, df=6	Two-tailed		P = 0.2039, ns<br>P = 0.1320, ns
			Hypothalamus / Half brain (ratio)	male	P42	WT, HT	4, 4	WT = 0.09908 ± 0.01118<br>HT = 0.1081 ± 0.006365	Student's t-test		P = 0.5113, ns	t=0.6981, df=6	Two-tailed		P = 0.9988, ns<br>P = 0.9880, ns
			Amygdala / Half brain (ratio)	male	P42	WT, HT	4, 4	WT = 0.03927 ± 0.0008886<br>HT = 0.03938 ± 0.001191	Student's t-test		P = 0.9457, ns	t=0.07101, df=6	Two-tailed		P = 0.1710, ns<br>P = 0.4710, ns
	c	Cortex Thickness	mm	male	P42	WT, HT	6(4), 6(4)	WT = 1.193 ± 0.02911<br>HT = 1.217 ± 0.05647	Mann-Whitney test		P = 0.3095, ns	U = 11	Two-tailed		P = 0.0077, **<br>P = 0.4006, ns
	d	NeuN staining	Fraction (ratio)	male	P42	WT, HT	6(4), 8(4)	2-WAY ANOVA			Interaction :: P = 0.9692, DF=9<br>Layer :: P < 0.0001, DF = 9<br>Genotype :: P = 0.0441, DF = 1				
			ea / mm2	male	P42	WT, HT	6(4), 8(4)	WT = 786.3 ± 78.9<br>HT = 728.1 ± 40.8	Mann-Whitney test		P = 0.1812, ns	U = 13	Two-tailed	P = 0.0121, *	P = 0.1053, ns<br>P = 0.0663, ns
	e	Cux1 staining	Fraction (ratio)	male	P42	WT, HT	6(2), 8(4)	2-WAY ANOVA			Interaction :: P = 0.0001, DF=9<br>Layer :: P < 0.0001, DF = 9<br>Genotype :: P = 0.7143, DF = 1				
								Sidak's Multiple Copmarison	1	WT vs HT	P = 0.0704, ns				
									2	WT vs HT	P = 0.0441, *				
									3	WT vs HT	P = 0.4693, ns				
									4	WT vs HT	P = 0.0006, ***				
									5	WT vs HT	P = 0.4980, ns				
									6	WT vs HT	P > 0.9999, ns				
									7	WT vs HT	P > 0.9999, ns				
									8	WT vs HT	P = 0.9990, ns				

									9	WT vs HT	P = 0.9984, ns				
									10	WT vs HT	P > 0.9999, ns				
			ea / mm2	male	P42	WT, HT	6(2), 8(4)	WT = 366.43 ± 57.797<br>HT = 439.17 ± 42.279	Students' t-test		P = 0.3178, ns	t=1.042, df=12	Two-tailed	P = 0.8716, ns	P = 0.3976, ns<br>P = 0.6043, ns
	f	FoxP2 staining	Fraction (ratio)	male	P42	WT, HT	6(3), 7(3)	2-WAY ANOVA		Interaction :: P = 0.3504, DF=9<br>Layer :: P < 0.0001, DF = 9<br>Genotype :: P = 0.0591, DF = 1					
			ea / mm2	male	P42	WT, HT	6(3), 7(3)	WT = 145.54 ± 19.306<br>HT = 153.97 ± 25.729	Students' t-test		P = 0.8037, ns	t=0.2546, df=11	Two-tailed		P = 0.9511, ns<br>P = 0.9232, ns
S3	b	Lateral Ventrical, Area	mm2	male	P42	WT, HT	4, 4	2-WAY ANOVA		Interaction :: P = 0.0163, DF=12<br>Axis :: P < 0.0001, DF = 12<br>Genotype :: P = 0.0837, DF = 1					
								Sidak's Multiple Copmarison	1.1	WT vs HT	P = 0.9996, ns				
									0.86	WT vs HT	P = 0.9999, ns				
									0.5	WT vs HT	P > 0.9999, ns				
									0.14	WT vs HT	P = 0.9652, ns				
									-0.1	WT vs HT	P = 0.0107, *				
									-0.22	WT vs HT	P = 0.0095, **				
									-0.46	WT vs HT	P = 0.4046, ns				
									-0.94	WT vs HT	P = 0.3918, ns				
									-1.22	WT vs HT	P = 0.9892, ns				
									-1.46	WT vs HT	P > 0.9999, ns				
									-1.7	WT vs HT	P = 0.9998, ns				
									-1.94	WT vs HT	P = 0.9998, ns				
									-2.18	WT vs HT	P > 0.9999, ns				
	c	Thrid Ventricle, Area	mm2	male	P42	WT, HT	4, 4	2-WAY ANOVA		Interaction :: P = 0.0344, DF=12<br>Axis :: P < 0.0001, DF = 12<br>Genotype :: P = 0.8082, DF = 1					
	d	Brain, Area	mm2	male	P42	WT, HT	4, 4	2-WAY ANOVA		Interaction :: P = 0.1695, DF=12<br>Axis :: P < 0.0001, DF = 12<br>Genotype :: P = 0.8806, DF = 1					
	e	Corpus Callosum, Area	mm2	male	P42	WT, HT	4, 4	2-WAY ANOVA		Interaction :: P < 0.0295, DF=12<br>Axis :: P < 0.0001, DF = 12<br>Genotype :: P = 0.0866, DF = 1					
								Sidak's Multiple Copmarison	1.1	WT vs HT	P = 0.9915, ns				
									0.86	WT vs HT	P = 0.9994, ns				
									0.5	WT vs HT	P > 0.9999, ns				
									0.14	WT vs HT	P > 0.9999, ns				
									-0.1	WT vs HT	P > 0.9999, ns				
									-0.22	WT vs HT	P > 0.9999, ns				
									-0.46	WT vs HT	P = 0.9996, ns				
									-0.94	WT vs HT	P = 0.8783, ns				
									-1.22	WT vs HT	P = 0.9291, ns				
									-1.46	WT vs HT	P = 0.9915, ns				
									-1.7	WT vs HT	P = 0.7419, ns				
									-1.94	WT vs HT	P = 0.0108, *				
									-2.18	WT vs HT	P = 0.0029, **				
	h	Sholl analysis, apical	ea	male	P42	WT, HT	13(2), 13(3)	2-WAY ANOVA		Interaction :: P = 0.0006, DF=16<br>Time :: P < 0.0001, DF = 16<br>Genotype :: P = 0.4928, DF = 1					
								Sidak's Multiple Copmarison	0	WT vs HT	P > 0.9999, ns				
									100	WT vs HT	P = 0.9914, ns				
									200	WT vs HT	P = 0.0124, *				
									300	WT vs HT	P = 0.5166, ns				
									400	WT vs HT	P = 0.0174, *				
									500	WT vs HT	P = 0.9989, ns				
									600	WT vs HT	P = 0.9997, ns				
									700	WT vs HT	P > 0.9999, ns				
									800	WT vs HT	P > 0.9999, ns				
									900	WT vs HT	P > 0.9999, ns				
									1000	WT vs HT	P > 0.9999, ns				
									1100	WT vs HT	P > 0.9999, ns				
									1200	WT vs HT	P = 0.9967, ns				
									1300	WT vs HT	P = 0.9806, ns				
									1400	WT vs HT	P = 0.9997, ns				
									1500	WT vs HT	P > 0.9999, ns				
									1600	WT vs HT	P > 0.9999, ns				
		Sholl analysis, basal	ea	male	P42	WT, HT	13(2), 13(3)	2-WAY ANOVA		Interaction :: P = 0.8643, DF=16<br>Time :: P < 0.0001, DF = 16<br>Genotype :: P = 0.7508, DF = 1					
		Sholl analysis, Total	ea	male	P42	WT, HT	13(2), 13(3)	2-WAY ANOVA		Interaction :: P = 0.1433, DF=16<br>Time :: P < 0.0001, DF = 16<br>Genotype :: P = 0.3442, DF = 1					
S4	a	Open field test, 100 lux	Distance moved (cm)	Male	P60~90	WT, HT	26, 27	2-WAY ANOVA		Interaction :: P = 0.0335, DF=5<br>Time :: P < 0.0001, DF = 5<br>Genotype :: P = 0.1257, DF = 1					
								Sidak's Multiple Copmarison	10	WT vs HT	P > 0.9999, ns				
									20	WT vs HT	P = 0.7798, ns				
									30	WT vs HT	P = 0.7531, ns				
									40	WT vs HT	P = 0.0251, *				
									50	WT vs HT	P = 0.9993, ns				
									60	WT vs HT	P = 0.3999, ns				
			Total Distance moved (cm)	male	P60-P90	WT, HT	26, 27	WT = 8856 ± 426.5<br>HT = 8050 ± 299	Mann-Whitney test		P = 0.2686, ns	U = 288	Two-tailed	P = 0.0265, *<br>P = 0.7706, ns	P = 0.1189, ns<br>P = 0.5615, ns
			Time in center (s)	male	P60-P90	WT, HT	26, 27	WT = 249.8 ± 40.25<br>HT = 333.7 ± 49.6	Mann-Whitney test		P = 0.1431, ns	U = 268	Two-tailed	P = 0.0003, ***<br>P = 0.0037, **	P = 0.0006, ***<br>P = 0.0012
	b	Open field test, 0 lux	Distance moved (cm)	Male	P60~90	WT, HT	13, 11	2-WAY ANOVA		Interaction :: P = 0.9880, DF=5<br>Time :: P < 0.0001, DF = 5<br>Genotype :: P = 0.0385, DF = 1					
								Sidak's Multiple Copmarison	10	WT vs HT	P = 0.9192, ns				
									20	WT vs HT	P = 0.9026, ns				
									30	WT vs HT	P = 0.9950, ns				
									40	WT vs HT	P = 0.7241, ns				
									50	WT vs HT	P = 0.9996, ns				
									60	WT vs HT	P = 0.9140, ns				
			Total Distance moved (cm)	male	P60-P90	WT, HT	13, 11	WT = 21302 ± 1010<br>HT = 19751 ± 1271	Student's t-test		P = 0.3437, ns	t=0.9678 df=22	Two-tailed	P = 0.9346, ns<br>P = 0.5222, ns	P = 0.9227, ns<br>P = 0.4058, ns
			Time in center (s)	male	P60-P90	WT, HT	13, 11	WT = 339.8 ± 36.19<br>HT = 250.7 ± 33.11	Student's t-test		P = 0.0873, ns	t=1.79 df=22	Two-tailed	P = 0.1087, ns<br>P = 0.7345, ns	P = 0.0530, ns<br>P = 0.9207, ns
	c	EPM	Time in area (s)	male	P60~90	WT, HT	16, 12	2-WAY ANOVA		Interaction :: P = 0.9297, DF=1<br>Arm :: P < 0.0001, DF = 1<br>Genotype :: P = 0.2894, DF = 1					
								Tukey's multiple comparisons test		Opened: WT vs. Closed: WT	P < 0.0001, ***				
										Opened: HT vs. Closed: HT	P < 0.0001, ***				
	d	Light Dark	Time in light box (s)	male	P60-P90	WT, HT	12, 14	WT = 69.43 ± 9.959<br>HT = 46.51 ± 11.32	Mann-Whitney test		P = 0.0596, ns	U = 47	Two-tailed	P = 0.8545, ns<br>P = 0.0377, *	P = 0.9822, ns<br>P = 0.0163, *
	e	Novel Object Recognition Test	Exploration Time (s)	Male	P60~90	WT, HT	12, 9	2-WAY ANOVA		Interaction :: P = 0.3346, DF=1<br>Object type :: P = 0.0228, DF = 1<br>Genotype :: P = 0.0016, DF = 1					
								Tukey's multiple comparisons test		Familiar:WT vs. Familiar:HT	P = 0.3305, ns				
										Familiar:WT vs. Novel:WT	P = 0.0668, ns				
										Familiar:WT vs. Novel:HT	P = 0.8854, ns				
										Familiar:HT vs. Novel:WT	P = 0.0012, **				
										Familiar:HT vs. Novel:HT	P = 0.7928, ns				
										Novel:WT vs. Novel:HT	P = 0.0184, *				
			Novel object sniffing index (N / N + F)	male	P60-P90	WT, HT	12, 9	WT = 0.6238 ± 0.02729<br>HT = 0.6301 ± 0.05388	Student's t-test		P = 0.9117, ns	t=0.2732 df=20	Two-tailed	P = 0.9562, ns<br>P = 0.7232, ns	P = 0.9969, ns<br>P = 0.8790, ns
S8	a	mPFC Prelimbic cortex Layer V, mEPSC	Frequency (Hz)	Male	P42	WT, HT	12 (3), 14 (3)	WT = 2.305 ± 0.3243<br>HT = 3.483 ± 0.8267	Mann-Whitney test		P = 0.7810, ns	U = 78	Two-tailed	P = 0.7636, ns<br>P = 0.1842, ns	P = 0.8464, ns<br>P = 0.0072, **
			Amplitude (pA)	Male		WT, HT	12 (3), 14 (3)	WT = 13.38 ± 0.6538<br>HT = 13.99 ± 0.768	Student's t-test		P = 0.5564, ns	t=0.5966 df=24	Two-tailed	P = 0.5411, ns<br>P = 0.0865, ns	P = 0.6662, ns<br>P = 0.1362, ns
	b	mPFC Prelimbic cortex Layer V, mIPSC	Frequency (Hz)	Male	P56	WT, HT	11 (3), 14 (3)	WT = 4.123 ± 0.4113<br>HT = 4.563 ± 0.5292	Student's t-test		P = 0.5359, ns	t=0.6284 df=23	Two-tailed	P = 0.7511, ns<br>P = 0.8139, ns	P = 0.6273, ns<br>P = 0.7970, ns
			Amplitude (pA)	Male		WT, HT	11 (3), 14 (3)	WT = 42.18 ± 3.615<br>HT = 38.35 ± 2.566	Student's t-test		P = 0.3838, ns	t=0.8879 df=23	Two-tailed	P = 0.7683, ns<br>P = 0.7525, ns	P = 0.7723, ns<br>P = 0.7973, ns
	c	mPFC Layer VI, mEPSC	Frequency (Hz)	male	P56	WT, HT	13(3), 14(3)	WT = 1.909 ± 0.9296<br>HT = 1.881 ± 0.6878	Students' t-test		P = 0.9294, ns	t=0.08950 df=25	Two-tailed	P = 0.4053, ns<br>P = 0.2809, ns	P = 0.2406, ns<br>P = 0.2897, ns
			Amplitude (pA)	male		WT, HT	13(3), 14(3)	WT = 16.25 ± 4.176<br>HT = 16.84 ± 2.739	Students' t-test		P = 0.6659, ns	t=0.4369, df=25	Two-tailed	P = 0.3152, ns<br>P = 0.5363, ns	P = 0.1138, ns<br>P = 0.4609, ns
	d	ACC Layer II/III, mEPSC	Frequency (Hz)	male	P56	WT, HT	16(3), 14(3)	WT = 1.016 ± 0.5803<br>HT = 1.109 ± 0.9861	Mann-Whitney test		P = 0.7277, ns	U = 103	Two-tailed	P = 0.6170, ns<br>P = 0.2130, ns	P = 0.4538, ns<br>P = 0.0096, **
			Amplitude (pA)	male		WT, HT	16(3), 14(3)	WT = 35.95 ± 6.087<br>HT = 34.89 ± 5.639	Students' t-test		P = 0.6238, ns	t=0.4959, df=28	Two-tailed	P = 0.2786, ns<br>P = 0.6731, ns	P = 0.3552, ns<br>P = 0.3974, ns
	e	ACC Layer V, mEPSC	Frequency (Hz)	male	P56	WT, HT	17(3), 13(3)	WT = 1.615 ± 1.174<br>HT = 1.915 ± 1.150	Mann-Whitney test		P = 0.3571, ns	U = 88	Two-tailed	P = 0.1168, ns<br>P = 0.1086, ns	P = 0.0062, **<br>P = 0.0998, ns
			Amplitude (pA)	male		WT, HT	17(3), 13(3)	WT = 14.05 ± 2.716<br>HT = 14.50 ± 3.722	Students' t-test		P = 0.7043, ns	t=0.3835, df=28	Two-tailed	P = 0.1303, ns<br>P = 0.2565, ns	P = 0.0587, ns<br>P = 0.2223, ns
	f	mPFC Layer II/III, sIPSC	Frequency (Hz)	male	P56	WT, HT	13(3), 14(3)	WT = 3.109 ± 2.052<br>HT = 3.882 ± 1.481	Mann-Whitney test		P = 0.0945, ns	U = 56	Two-tailed	P = 0.0538, ns<br>P = 0.2364, ns	P = 0.0180, *<br>P = 0.0119, *
			Amplitude (pA)	male		WT, HT	13(3), 14(3)	WT = 72.43 ± 14.28<br>HT = 70.49 ± 18.46	Students' t-test		P = 0.7635, ns	t=0.3041, df=25	Two-tailed	P = 0.8676, ns<br>P = 0.1415, ns	P = 0.9192, ns<br>P = 0.2222, ns
	g	Quantitative measurement of PSD using electron microscopy	GABA+ Terminal / 1000 μm2	male	P56	WT, HT	3 (96 EM), 3 (96 EM)	WT = 199.9 ± 4.625<br>HT = 205.2 ± 5.161	Students' t-test		P = 0.2529, ns	t=1.335, df=4	Two-tailed		P = 0.8781, ns
	h	IHC, using anti-GABA antibody	GABA cells / mm2	male	P56	WT, HT	3, 3	WT = 76.01 ± 5.193<br>HT = 79.35 ± 9.875	Students' t-test		P = 0.5813, ns	t=0.5892, df=5	Two-tailed	P = 0.8864, ns<br>P = 0.9584, ns	
S10	a	Body Weight	g	Male	P3~P53	WT (VEH), WT (FLX), HT (VEH), HT (FLX)	30, 42, 18, 30	Mixed effects analysis		Age :: P < 0.0001, ***, DF = 5<br>Drug :: P = 0.0147, *, DF = 1<br>Genotype :: P < 0.0001, ***, DF = 1<br>Age X Genotype :: P < 0.0001, ***, DF = 5<br>Age X Drug :: P < 0.0001, ***, DF = 5<br>Drug X Genotype :: P = 0.1709, ns, DF = 1<br>Age X Drug X Genotype :: P = 0.4287, ns, DF = 5					
	b	Open field test, 100 lux	Distance moved (m)	male	P60-P90	WT (VEH), WT (FLX), HT (VEH), HT (FLX)	15, 25, 18, 14	3-WAY ANOVA		Time :: P < 0.0001, ***, DF = 5<br>Drug :: P = 0.0189, *, DF = 1<br>Genotype :: P = 0.4584, ns, DF = 1<br>Time X Drug :: P = 0.5456, ns, DF = 5<br>Time X Genotype :: P = 0.0178, *, DF = 5<br>Drug X Genotype :: P = 0.4749, ns, DF = 1<br>Time X Drug X Genotype :: P = 0.2886, ns, DF = 5					
	c		Total Distance moved (m)	male	P60-P90	WT (VEH), WT (FLX), HT (VEH), HT (FLX)	15, 25, 18, 14	2-WAY ANOVA		Interaction :: P = 0.3949, ns, DF = 1<br>Drug :: P = 0.0144, *, DF = 1<br>Genotype :: P = 0.5630, ns, DF = 1					
	d		Time in Center (s)	male	P60-P90	WT (VEH), WT (FLX), HT (VEH), HT (FLX)	15, 25, 18, 14	2-WAY ANOVA		Interaction :: P = 0.9910, ns, DF = 1<br>Drug :: P = 0.5038, ns, DF = 1<br>Genotype :: P = 0.0011, **, DF = 1					
S11	a	Direct interaction test	Time sniffing time (s)	Male	P100-P200	WT (VEH), WT (FLX), HT (VEH), HT (FLX)	11 (22), 11 (22), 10 (10), 10 (20)	2-WAY ANOVA		Interaction :: P = 0.6065, DF=1<br>Drug :: P = 0.3113, DF = 1<br>Genotype :: P < 0.0001, DF = 1					
	b	Self grooming test	Self grooming time (s)	Male	P100-P200	WT (VEH), WT (FLX), HT (VEH), HT (FLX)	18, 18, 18, 18	2-WAY ANOVA		Interaction :: P = 0.4954, DF=1<br>Drug :: P = 0.1109, DF = 1<br>Genotype :: P < 0.0001, DF = 1					
	c	Open filed test	moved distance (m)	Male	P100-P200	WT (VEH), WT (FLX), HT (VEH), HT (FLX)	24, 24, 20, 19	3-WAY ANOVA		Time :: P < 0.0001, DF=5<br>Drug :: P < 0.0001, DF = 1<br>Genotype :: P = 0.0740, DF = 1					
	b	Open filed test	Total moved distance (m)	Male	P100-P200	WT (VEH), WT (FLX), HT (VEH), HT (FLX)	24, 24, 20, 19	2-WAY ANOVA		Interaction :: P = 0.4100, DF=1<br>Drug :: P < 0.0001, DF = 1<br>Genotype :: P = 0.0740 DF = 1					
S13	a	Western blot: HDAC4	mPFC P1 fraction relative expression levels	Male	P120	WT (VEH), WT (FLX), HT (VEH), HT (FLX)	3, 3, 3, 3	2-WAY ANOVA		Interaction :: P = 0.0018, DF=1<br>Drug :: P = 0.0249, DF = 1<br>Genotype :: P = 0.0510, DF = 1					
								Tukey's Multiple Copmarison		VEH:WT vs. VEH:HT	P = 0.0054, **				
										VEH:WT vs. FLX:WT	P = 0.5872, ns				
										VEH:WT vs. FLX:HT	P = 0.9871, ns				
										VEH:HT vs. FLX:WT	P = 0.0300, *				
										VEH:HT vs. FLX:HT	P = 0.0037, **				
										FLX:WT vs. FLX:HT	P = 0.4169, ns				
	b	Western blot: SYNGAP	mPFC P2 fraction relative expression levels	Male	P120	WT (VEH), WT (FLX), HT (VEH), HT (FLX)	3, 3, 3, 3	2-WAY ANOVA		Interaction :: P = 0.0454, DF=1<br>Drug :: P = 0.0288, DF = 1<br>Genotype :: P = 0.0130, DF = 1					
								Tukey's Multiple Copmarison		VEH:WT vs. VEH:HT	P = 0.0185, *				
										VEH:WT vs. FLX:WT	P = 0.9966, ns				
										VEH:WT vs. FLX:HT	P = 0.9818, ns				
										VEH:HT vs. FLX:WT	P = 0.0140, *				
										VEH:HT vs. FLX:HT	P = 0.0306, *				
										FLX:WT vs. FLX:HT	P = 0.9370, ns				
	c	Western blot: ARC	mPFC P2 fraction relative expression levels	Male	P120	WT (VEH), WT (FLX), HT (VEH), HT (FLX)	3, 3, 3, 3	2-WAY ANOVA		Interaction :: P = 0.3209, DF=1<br>Drug :: P = 0.1753, DF = 1<br>Genotype :: P = 0.3439, DF = 1					
S14	a	Western blot: ARID1B	Whole brain relative expression levels	Male	P120	WT (VEH), WT (FLX), HT (VEH), HT (FLX)	3, 3, 3, 3	2-WAY ANOVA		Interaction :: P = 0.7950, DF=1<br>Drug :: P = 0.9271, DF = 1<br>Genotype :: P = 0.0007, DF = 1					
			P1 fraction relative expression levels	Male	P120	WT (VEH), WT (FLX), HT (VEH), HT (FLX)	3, 3, 3, 3	2-WAY ANOVA		Interaction :: P = 0.6290, DF=1<br>Drug :: P = 0.7271, DF = 1<br>Genotype :: P = 0.0065, DF = 1					
	b	Western blot: β-catenin	Whole brain relative expression levels	Male	P120	WT (VEH), WT (FLX), HT (VEH), HT (FLX)	3, 3, 3, 3	2-WAY ANOVA		Interaction :: P = 0.4451, DF=1<br>Drug :: P = 0.2806, DF = 1<br>Genotype :: P = 0.2033, DF = 1					
			P1 fraction relative expression levels	Male	P120	WT (VEH), WT (FLX), HT (VEH), HT (FLX)	3, 3, 3, 3	2-WAY ANOVA		Interaction :: P = 0.5240, DF=1<br>Drug :: P = 0.2865, DF = 1<br>Genotype :: P = 0.0484, DF = 1					
	c	Western blot: pβ-catenin S552	Whole brain relative expression levels	Male	P120	WT (VEH), WT (FLX), HT (VEH), HT (FLX)	3, 3, 3, 3	2-WAY ANOVA		Interaction :: P = 0.1478 DF=1<br>Drug :: P = 0.9577, DF = 1<br>Genotype :: P = 0.2220, DF = 1					
			P1 fraction relative expression levels	Male	P120	WT (VEH), WT (FLX), HT (VEH), HT (FLX)	3, 3, 3, 3	2-WAY ANOVA		Interaction :: P = 0.7083, DF=1<br>Drug :: P = 0.4372, DF = 1<br>Genotype :: P = 0.1532, DF = 1					
	d	Western blot: pβ-catenin S675	Whole brain relative expression levels	Male	P120	WT (VEH), WT (FLX), HT (VEH), HT (FLX)	3, 3, 3, 3	2-WAY ANOVA		Interaction :: P = 0.7652 DF=1<br>Drug :: P = 0.7827 DF = 1<br>Genotype :: P = 0.1163, DF = 1					
			P1 fraction relative expression levels	Male	P120	WT (VEH), WT (FLX), HT (VEH), HT (FLX)	3, 3, 3, 3	2-WAY ANOVA		Interaction :: P = 0.1221, DF=1<br>Drug :: P = 0.0140, DF = 1<br>Genotype :: P = 0.6791, DF = 1					
